# Comparison of Toxicity of Heavy Metals and Rare Earth Elements in Zebrafish Larvae and *Daphnia magna* Neonates Using a Phenomics Approach with Multiple Locomotor Activity Endpoints

**DOI:** 10.3390/toxics14100862

**Published:** 2026-09-28

**Authors:** Basit Ali Khan, Minh-Quan Tran, Muhammad Baqir Khan, Chung-Der Hsiao

**Affiliations:** 1 Department of Biomedical Engineering, Chung Yuan Christian University, Taoyuan 320314, Taiwan; basitmbio@gmail.com (B.A.K.); minhquan0141@gmail.com (M.-Q.T.); 2 Department of Bioscience Technology, Chung Yuan Christian University, Taoyuan 320314, Taiwan; baqirkhanmmc@gmail.com; 3 Department of Chemistry, Chung Yuan Christian University, Taoyuan 320314, Taiwan; 4 Research Center for Aquatic Toxicology and Pharmacology, Chung Yuan Christian University, Taoyuan 320314, Taiwan

**Keywords:** heavy metals, rare earth elements, zebrafish, *Daphnia magna*, phenomics

## Abstract

Heavy metals (HMs) and rare earth elements (REEs) persist as environmental contaminants that accumulate in aquatic ecosystems and pose risks to aquatic organisms. Although the toxicity of HMs is well-established, the ecological and biological impacts of REEs remain comparatively underexplored, despite their expanding industrial use. In this study, zebrafish (*Danio rerio*) larvae and *Daphnia magna* neonates were employed as complementary aquatic models to comparatively assess the toxicological effects of multiple metal groups, leveraging their rapid development and suitability for high-throughput behavioral analysis. Both species were exposed to 17 HMs and 16 REEs across concentration ranges defined by species-specific guidelines to determine LC_50_ values, followed by 24 h behavioral assessments at sublethal concentrations, and employing optimized, high-throughput protocols. Thirteen behavioral endpoints were analyzed to comprehensively characterize locomotor activity, spatial exploration, and movement complexity. Multivariate behavioral patterns were evaluated using principal component analysis and heatmap clustering. The results revealed pronounced species- and metal-specific toxicity profiles, with several REEs inducing behavioral effects comparable to or exceeding those of certain HMs. Notably, behavioral responses differed between zebrafish larvae and *Daphnia magna* for the same compounds, highlighting species-specific sensitivity and underscoring the value of multi-species assessment for environmental risk evaluation.

## 1. Introduction

Heavy Metals (HMs) are naturally occurring elements characterized by high atomic weights and densities. Their extensive use in industrial, agricultural, and technological sectors has led to their ubiquitous presence in the environment [1]. HMs, such as chromium (Cr), cobalt (Co), iron (Fe), manganese (Mn), nickel (Ni), vanadium (V), are especially concerning because of their high solubility and bioavailability in aquatic environments [2]. The rapid growth of industrialization and urbanization has led to a substantial increase in the release of these compounds into water sources, posing serious risks to environmental and human health [3]. Chronic or acute exposure to HMs can lead to various harmful effects, such as neurotoxicity, cancer risk, and interference with metabolic functions in both humans and wildlife [4]. HMs, due to their persistence and bioaccumulation, can harm non-target aquatic species, disrupt ecological balance, and pose a threat to biodiversity [5,6]. In aquatic environments, organisms such as *Daphnia magna* and zebrafish (*Danio rerio*) are often exposed to complex mixtures of HMs. These exposures usually occur at sublethal levels, which might not cause immediate mortality but can result in notable behavioral and physiological alterations [7,8]. Traditional toxicity assessments have primarily focused on outcomes such as mortality and developmental defects. However, recent research highlights the importance of behavioral endpoints as sensitive markers of neurotoxicity and ecological impact risk.

Rare Earth Elements (REEs) occur naturally in the Earth’s crust and are also present in aquatic environments, including oceans, rivers, and lakes, at concentrations ranging from nanograms to micrograms per liter [9]. REEs are classified on the basis of atomic properties and geochemical characteristics into light rare earth elements (LREEs) and heavy rare earth elements (HREEs). REEs consist of 17 chemically similar metals, including 15 lanthanide elements, cerium (Ce), dysprosium (Dy), erbium (Er), europium (Eu), gadolinium (Gd), holmium (Ho), lanthanum (La), lutetium (Lu), neodymium (Nd), praseodymium (Pr), promethium (Pm), samarium (Sm), terbium (Tb), thulium (Tm), and ytterbium (Yb), along with scandium (Sc) and yttrium (Y). These metals are increasingly entering aquatic systems through mining, high-tech manufacturing, and electronic waste disposal [10,11]. Their increasing use in modern technology raises concerns about their environmental impact. Multiple studies have shown that REEs can have notable biological and ecological effects, including inhibiting growth, causing developmental malformations, immobilizing organisms, and altering biomarker responses [11]. These effects have been reported in a variety of aquatic species, including *Aliivibrio fischeri*, *Crassostrea gigas*, *Fucus vesiculosus*, *Raphidocelis subcapitata*, *Ulva lactuca*, *Brachionus calyciflorus*, *Daphnia magna*, and *Danio rerio* [12,13,14,15]. *Daphnia magna* is a standard and sensitive aquatic model for heavy metal toxicity assessment, with OECD-guided 48 h acute bioassays demonstrating strong responses to metals such as copper (Cu), zinc (Zn), cadmium (Cd), lead (Pb), and mercury (Hg) [16]. These metals cause mortality, locomotor impairment, oxidative stress, and metal-specific detoxification responses, including antioxidant enzyme activation, metallothionein binding, and extensive transcriptomic alterations at sublethal exposures [17,18,19]. Owing to its high sensitivity and good concordance with fish and other aquatic bioassays, *Daphnia magna* is widely used as a sentinel species for evaluating metal-induced ecological risk in aquatic environments [16]. In parallel, in zebrafish larvae, exposure to Eu or Sm impairs swimming behavior, reduces growth, decreases heart rate, causes morphological deformities, and changes gene expression related to oxidative phosphorylation and apoptosis [20]. Beyond immediate lethality, long-term effects vary widely across species, with calcifying organisms such as sea urchins being more sensitive, possibly because of competition for calcium-binding sites [21]. Overall, these results emphasize the importance of including REEs in aquatic toxicology evaluations, given that their environmental interactions and toxicity profiles differ significantly from those of more extensively studied HMs.

*Daphnia magna* and zebrafish larvae are widely used model organisms in ecotoxicological studies, as both share key characteristics, including rapid development, transparent bodies, and ease of laboratory maintenance, which make them highly suitable for toxicity screening and environmental risk assessment [8,22,23]. In this regard, zebrafish are widely used in research owing to their distinctive behaviors, suitability for rodent studies, and capacity to adapt to novel environments [24]. They serve as a valuable model for examining how pharmacological compounds affect behavior, with findings often applicable to mammals and humans [25,26]. Advances in technology have made zebrafish ideal for investigating neural control of behaviors such as feeding, anxiety, and circadian rhythms [27,28]. Their small size, low maintenance, and high reproductive rate, producing 200–300 transparent eggs at once, also make them ideal for biomedical research, including developmental, toxicological, and genetic studies [25]. *Daphnia magna* are commonly used in environmental and toxicological research due to their sensitivity to pollutants, short life cycle, and simple laboratory cultivation [29]. They serve as an important model for assessing the impacts of HMs and REEs in aquatic environments, with results frequently informing ecological risk evaluations [30,31]. Their small size, transparent bodies, and high reproductive rate make them excellent models for studying physiological and developmental responses to chemical exposure [31]. Diverse behavioral parameters, such as total distance traveled, burst count, rotation average speed, average angular velocity, meandering, swimming ratio, thigmotaxis, time spent in the central area, rapid movement time, fractal dimension, entropy, and freezing time, provide detailed insights into how toxicants affect sublethal aspects of locomotor and exploratory behaviors [32]. These endpoints are particularly significant because they are directly linked to essential survival behaviors, including feeding, predator avoidance, and social interaction.

HMs and REEs are present in aquatic environments at concentrations that vary widely depending on the element, its sources (both natural and human-related), and the water’s physicochemical properties [33]. These factors, including pH, redox conditions, temperature, ionic strength, salinity, and dissolved organic matter, affect metal speciation, solubility, mobility, and bioavailability [34]. HMs can exist at trace levels in clean waters or at much higher levels in waters receiving industrial, mining, agricultural, or urban discharges [35]. Similarly, REEs are typically found at trace-to-low µg/L levels in surface waters, but higher concentrations can occur near mining sites, industrial activities, or electronic waste sites [36]. As a result, environmental concentrations differ significantly among elements and contamination contexts. To offer an environmental perspective for the concentrations examined here, the ranges of HMs and REEs in natural and contaminated aquatic systems are detailed in Table 1.

In this study, we systematically investigated the acute and sublethal neurobehavioral toxicity of 17 HMs (HMs) and 16 REEs using a dual-species aquatic model comprising zebrafish larvae and *Daphnia magna*. Acute toxicity and sublethal behavioral responses were assessed using a combination of LC_50_ determination and high-resolution, multi-endpoint behavioral phenomics. Multivariate analyses, including principal component analysis (PCA) and hierarchical clustering, were further applied to identify patterns in behavioral responses across elements and species [37,38]. The specific objectives were to (i) determine and compare the acute toxicity of the tested HMs and REEs by establishing LC_50_ values across multiple concentrations (0.1–100 mg/L), with zebrafish larvae exposed for 96 h and *Daphnia magna* for 48 h; (ii) characterize sublethal behavioral effects at a standardized exposure concentration using high-resolution, multi-endpoint behavioral phenomics; and (iii) identify species- and element-specific behavioral response patterns through multivariate analyses, including principal component analysis (PCA) and hierarchical clustering. This integrative approach allowed us to characterize species and metal specific behavioral toxicity profiles, assess whether neurobehavioral disturbances occur independently of lethality, and compare toxicity rankings between vertebrate and invertebrate models, establishing behavioral phenomics as a sensitive tool for ecological risk assessment of metal contaminants.

**Table 1:** Representative concentrations of the studied HMs and REEs reported in natural/relatively uncontaminated and contaminated or environmentally impacted aquatic systems.

Element	Element Group	Natural/Relatively Uncontaminated Water (µg/L)	Concentration in Contaminated Waters (µg/L)	References
Aluminum (Al)	HMs	~4	20.3–2615	[33]
Antimony (Sb)	HMs	1.45	15.66	[39]
Bismuth (Bi)	HMs	0.0127–0.0240	0.0121–0.0527	[40]
Chromium (Cr)	HMs	<10	2–100	[41]
Cobalt (Co)	HMs	0.0001	101	[42]
Gallium (Ga)	HMs	0.002–0.16	7.90–41.39	[43]
Iron (Fe)	HMs	40	200	[44]
Lithium (Li)	HMs	1	1700	[45]
Manganese (Mn)	HMs	~2.4	908.5	[33]
Molybdenum (Mo)	HMs	~2.6	802.4	[33]
Nickel (Ni)	HMs	0.67	590	[46]
Platinum (Pt)	HMs	0.04	0.41–0.71	[47]
Strontium (Sr)	HMs	~71.1	1236	[33]
Tin (Sn)	HMs	~0.1	3.5	[33]
Vanadium (V)	HMs	~1.7	528	[33]
Zinc (Zn)	HMs	~0.01	6060	[33]
Zirconium (Zr)	HMs	0.022	0.021	[48]
Cerium (Ce)	LREEs	0.22	152	[49]
Dysprosium (Dy)	HREEs	0.08	3.80	[49]
Erbium (Er)	HREEs	0.03	2.34	[49]
Europium (Eu)	LREEs	0.018	1.52	[49]
Gadolinium (Gd)	HREEs	0.028	7.22	[49]
Holmium (Ho)	HREEs	0.014	0.18	[49]
Lanthanum (La)	LREEs	0.10	140	[49]
Lutetium (Lu)	HREEs	0.006	0.21	[49]
Neodymium (Nd)	LREEs	0.095	52.0	[49]
Praseodymium (Pr)	LREEs	0.034	16.1	[49]
Terbium (Tb)	HREEs	0.006	0.88	[49]
Thulium (Tm)	HREEs	0.007	0.24	[49]
Samarium (Sm)	LREEs	0.054	6.91	[49]
Scandium (Sc)	HREEs	~0.06	31.34–50.81	[50]
Ytterbium (Yb)	HREEs	0.032	1.48	[49]
Yttrium (Y)	HREEs	~0.15–0.20	187–363.10	[50]

## 2. Materials and Methods

### 2.1. Overview of Experimental Design

Figure 1 illustrates the workflow for assessing the toxicity of HMs and REEs in zebrafish larvae and *Daphnia magna* neonates using a multi-endpoint behavioral phenomics method. Both model organisms were first maintained under standardized laboratory conditions and then exposed to 17 HM and 16 REEs at four concentrations (0.1 mg/L, 1 mg/L, 10 mg/L, and 100 mg/L) to determine LC_50_ values, using 96 h exposure for zebrafish larvae and 48 h exposure for *Daphnia magna* [51,52]. Following the acute toxicity assessment, sublethal concentrations (0.1 mg/L) were selected for behavioral testing. After exposure, the organisms were transferred to well plates and allowed to acclimate for 30 min, after which their activity was recorded under alternating light–dark cycles using the ZebraBox high-throughput tracking system. Locomotor trajectories were analyzed using both ViewPoint software (ViewPoint, 3.22.3.85, ViewPoint Life Sciences, Inc.: Civrieux, France, 2014, http://www.viewpointlifesciences.com, accessed on 21 September 2026) and (idtracker.ai, 6.0.12, https://idtracker.ai/latest/, accessed on 21 September 2026) to acquire accurate movement coordinates for each individual. Behavioral endpoints were quantified to capture sublethal effects on locomotion and exploratory behavior. The dataset was examined with multivariate statistical techniques, such as PCA and hierarchical clustering, to identify species-specific and metal-specific behavioral patterns. This comprehensive, high-resolution method allows for sensitive detection of neurobehavioral changes, supporting ecotoxicological risk assessment across various aquatic models Figure 1.

### 2.2. Zebrafish Larvae Rearing and Maintenance

For this study, we purchased PET-strain zebrafish from the local aquarium store and kept them in a recirculating aquatic system at 26.5 ± 1 °C, with water conductivity between 300 and 1500 µS/cm, pH maintained between 6.8 and 7.5, and a light–dark cycle of 14:10 h [8]. Before the experiments, adult fish were fed twice daily. For breeding, two males and one female were placed in a breeding tank overnight. A transparent divider separated the fish overnight, which was removed the following morning to allow for spawning, in accordance with our previous protocol [8,25,53]. Collected embryos were sanitized with methylene blue (0.0001%) as a fungicide until exposure began and incubated at 28 °C in E3 medium. The E3 medium was prepared with 5 mM NaCl, 0.17 mM KCl, 0.33 mM CaCl_2_, and 0.33 mM MgSO_4_ and used as the embryo-rearing and test medium. Embryos were checked daily to remove any dead individuals, and the medium was refreshed until 3 days post-fertilization (dpf). Larvae were kept in the same environmental conditions from 4 days post-fertilization.

### 2.3. Daphnia magna Rearing and Maintenance

In this study, we obtained *Daphnia magna* from the Freshwater Bioresource Center at National Chiayi University and maintained them under laboratory conditions for toxicity tests. A single clone was propagated parthenogenetically and grown in 12 L of medium under a 14:10 light–dark cycle at 20 ± 2 °C. The water had a pH of 6.7 and a conductivity of 300–1500 µS [8,54]. The daphnids were fed green algae, and the culture water was renewed every 2 days, with at least 80% of the volume replaced. Individuals were filtered to maintain uniform body size, and medium-sized daphnids (0.6–3.0 mm) were chosen for the experiments [55].

### 2.4. Chemical Preparation

This experiment involved 17 HMs and 16 REEs to evaluate their effects on zebrafish larvae and *Daphnia magna*; details are shown in Table 2. The metallic elements, in powder form with a purity of at least 98%, were obtained from Aladdin (Shanghai). Initially, these HMs and REEs were dissolved in ddH2O and diluted to the desired working solution for specific experiments. The solutions were stored at 25 °C to assess the behaviors of zebrafish larvae and *Daphnia magna*.

### 2.5. Acute Toxicity Test

After evaluating the quality of the zebrafish embryos, toxicity assays were conducted. First, 10 healthy embryos were placed in each 5.5 cm Petri dish. A 96 h acute exposure was performed, starting at 24 h post-fertilization (hpf) and continuing to 120 hpf [56]. The embryos were exposed to various nominal concentrations of HMs and REEs (0.1 mg/L, 1 mg/L, 10 mg/L, and 100 mg/L) in water, all maintained at 25 ± 0.5 °C. Each dish contained 20 mL of the test solution, following previous research [51,57]. Acute toxicity testing in zebrafish was conducted over a 96 h exposure period with reference to OECD Test Guideline No. 236 (Fish Embryo Acute Toxicity Test), during which embryo mortality was recorded at each concentration. Tests were performed in duplicate, with 10 embryos per treatment group, for a total of 20 embryos per concentration. This protocol aligns with standard guidelines for acute toxicity testing in zebrafish embryos, designed to assess toxicity and determine LC_50_ values. Acute toxicity testing of *Daphnia magna* neonates was conducted over a 48 h exposure period with reference to OECD Test Guideline No. 202 (*Daphnia* sp. Acute Immobilisation Test). In this test, ten neonates were placed in each 5.5 cm Petri dish for the control and treatment groups and exposed to the respective test concentrations under identical experimental conditions [52,58,59]. The temperature was maintained at 22 ± 2 °C, and mortality was recorded at the end of the exposure period to evaluate acute toxicity.

### 2.6. Exposure to HMs and REEs

In this experiment, zebrafish larvae at 4 dpf and *Daphnia magna* neonates were used to assess the impact of HM and REE exposure. Each trial included 55 zebrafish larvae and 55 *Daphnia magna* in the control group, while 30 individuals of each species were assigned to each treatment group [8]. For six-well plates, nine organisms were incubated. Following established protocols, each group was kept in a 9 cm Petri dish with 40 mL of deionized water (control) or a solution with a nominal concentration of 0.1 mg/L HMs and REEs (treatment) for 24 h. After exposure, control- and treatment-group zebrafish larvae and *Daphnia magna* were each placed in separate wells of a 48-well plate to measure locomotor activity. To minimize handling stress, a 30 min acclimation period was given in the recording device (ZebraBox, ViewPoint Life Sciences) before collecting behavioral data. This method enabled a consistent assessment of locomotor responses to HM and REE exposure across different model organisms. The concentrations and exposure conditions were selected based on prior toxicity data to evaluate sublethal effects in both species. An overview of the experimental design and the concentrations of HMs and REEs used is summarized in Table 1 and Figure 1.

### 2.7. Analysis of Automated Imaging of Swimming Behaviors

In this experiment, zebrafish larvae at 4 days post-fertilization and *Daphnia magna* neonates were exposed to HMs and REEs. Their locomotor behaviors were then analyzed using an automated high-throughput imaging system (ViewPoint, 3.22.3.85, ViewPoint Life Sciences, Inc., Civrieux, France, 2014, http://www.viewpointlifesciences.com, accessed on 21 September 2026) according to the protocol described [8]. Individual organisms were placed in 48-well plates with 800 µL of exposure medium per well and recorded under infrared light for 80 min. This session included alternating 10 min periods of light (visible light; 75% intensity) and dark (infrared light) between 9:00 and 16:00. Subsequently, the same videos were analyzed to determine burst counts and rotational movements. The activity of each zebrafish larva and *Daphnia magna* neonate was recorded every minute during the entire recording period for all three behavioral endpoints. For zebrafish larvae and *Daphnia magna* neonates, for a sample size of 24, the 48-well plates were divided into two sections to test two different HM and REE treatments simultaneously. Behavioral endpoints, including total distance traveled, burst count, and rotation count Supplementary Table S1, were measured at 1 min intervals for each individual, as previously described [8,25]. Movements were categorized as inactivity, cruising, and large movements based on velocity thresholds [44]. Swimming speed was classified into three categories: large movement (>20 mm/s), cruising movement (0.5–20 mm/s), and inactivity (<0.5 mm/s). For burst activity quantification, video-tracking settings were adjusted as follows: burst threshold = 20, freeze (no movement) threshold = 2, and detection sensitivity threshold = 20. Rotational movement, which is a sensitive indicator of locomotor activity, was also assessed. This behavior involves changes in body orientation in response to environmental cues such as food availability, light conditions, chemical stressors, or predator threats [8,25,60]. Clockwise and counterclockwise movements of zebrafish larvae were measured in millimeters, with a rotation defined as a movement exceeding 2 mm in diameter; movements below this threshold were excluded. The back angle for rotation detection was set at 60°. For all behavioral assay recordings, background subtraction was applied, and the detection threshold was set to 20 grayscale pixel intensity units, such that any pixel darker than this threshold was identified as part of the animal [25].

### 2.8. Locomotor Tracking Using idtracker.ai

To examine the locomotor behavior of zebrafish (*Danio rerio*) larvae and *Daphnia magna*, we used idtracker.ai, an advanced automated tracking system specifically designed to monitor multiple unmarked animals in video recordings [61]. This software uses a deep learning pipeline to accurately identify individuals and extract their trajectories, even in dense animal groups. In our study, we captured high-resolution videos using the ZebraBox system under controlled lighting and processed them with idtracker.ai. The software initially detected animals in each frame through background subtraction and image segmentation, then used a trained convolutional neural network to assign unique identities. Once tracking was complete, idtracker.ai provided precise time-series data, including the X- and Y-coordinates of each individual across all frames. These X-Y coordinates of zebrafish larvae and *Daphnia magna* are the primary data used to extract various behavioral endpoints.

### 2.9. Behavioral Endpoints

Once the locomotion recording protocol was finished, the analysis focused on extracting data from behavioral endpoints. The tracked X and Y coordinates were used to calculate various behavioral endpoints, including average speed, average angular velocity, meandering, swimming ratio, thigmotaxis, time spent in the central area, rapid movement time, fractal dimension, entropy, and freezing time, using established calculation methods Supplementary Table S1 [27,62,63]. These behavioral parameters are considered indicators of anxiety-like responses across various model organisms, such as mice, earthworms, and zebrafish [64,65,66]. This comprehensive approach enabled a detailed assessment of sublethal behavioral changes induced by HMs and REEs in these aquatic models.

### 2.10. Statistical Analysis

All statistical analyses and graphical visualizations were performed using GraphPad Prism (version 8; GraphPad Software, La Jolla, CA, USA). Behavioral parameters from treatment groups were evaluated in comparison with the corresponding control group. Data distribution was first examined using normality tests. Depending on the data characteristics, a two-way analysis of variance (ANOVA) was applied, followed by Dunnett’s multiple comparison test. For behavioral endpoints that did not meet parametric assumptions, one-way analysis of variance (ANOVA non-parametric statistical approaches were employed, including the Kruskal–Wallis test with Dunn’s multiple comparison correction. Results are expressed as mean ± standard error of the mean (SEM). Statistical significance was defined as * *p* < 0.05, ** *p* < 0.01, *** *p* < 0.001, and **** *p* < 0.0001.

### 2.11. PCA, Hierarchical Clustering, and Toxicity Ranking

To provide thorough phenomics characterization and to reduce dimensionality in complex behavioral datasets, multivariate analyses, including PCA, hierarchical clustering, and toxicity ranking, were conducted using all measured behavioral endpoints [66,67]. These methodologies are widely used in behavioral toxicology to combine various phenotypic responses and identify compound-specific patterns. We initially analyzed the raw behavioral data for both zebrafish larvae and *Daphnia magna* to get the mean values for each treatment group. The processed data were then exported in comma-separated values (.csv) format for multivariate analysis. PCA was used to illustrate the overall variance, grouping patterns, and interrelations among HMs and REEs according to their behavioral effect profiles. Using the ClustVis online platform (https://biit.cs.ut.ee/clustvis/, accessed on 17 March 2026), we performed hierarchical clustering and generated heatmaps of all behavioral endpoints. This enabled the systematic classification of compounds based on the similarity of their phenomics response. Heatmap endpoint responses were converted to numerical values to enable the quantitative evaluation and comparison of the behavioral toxicity of HMs and REEs. For each compound, all behavioral parameter values were combined to generate a composite toxicity index. This index provides a detailed evaluation of overall behavioral toxicity by consolidating endpoint-specific variations into a single ranking. The compounds were then categorized based on their total index scores, with higher scores indicating greater behavioral toxicity. The detailed methodology and toxicity ranking estimates are available in the Supplementary Table S18. Additionally, Spearman’s rank correlation analysis was used to assess the degree of similarity between the behavioral response patterns of zebrafish larvae and *Daphnia magna*. This enabled us to assess of the degree to similarity between the two species in their sensitivity to exposure to HMs and REEs.

## 3. Results

### 3.1. Acute Toxicity Test of Zebrafish Larvae and Daphnia magna After HM and REE Exposure

We conducted acute toxicity assays to compare the lethal effects of various HMs and REEs on zebrafish larvae and *Daphnia magna* Figure 2. Mortality data were subsequently used to determine median lethal concentrations (LC_50_). Toxicity responses showed different sensitivities depending on the metal or organism, with significant variations between compounds and species. Each curve represents a different HM or REE tested at designed concentrations. Most compounds exhibited a distinct sigmoidal dose–response curve, indicating that mortality increased with concentration. Variations in the steepness and horizontal shifts in these curves reflect differences in toxicity levels and species sensitivity. Notably, several HMs demonstrated acute toxicity that was similar to or higher than that of specific REEs.

Importantly, *Daphnia magna* exhibited higher sensitivity to most tested compounds compared with zebrafish larvae, highlighting clear species-specific differences in acute toxicity responses. Detailed LC_50_ values for both species are listed in Supplementary Table S2.

### 3.2. Total Distance Chronology of Zebrafish Larvae Following HM and REE Exposure

Zebrafish larvae showed higher locomotor activity during the dark phase than during the light phase across the experimental groups Supplementary Figure S1. Exposure to HMs and REEs altered total distance traveled in a compound-specific manner. During the light phase, Sb, Cr, Ce, Dy, and Ho significantly increased total distance traveled compared with the control group Figure 3A. During the dark phase, Sb, Cr, and Dy also significantly increased total distance traveled Figure 3B.

Several REEs, including Ho, Dy, Tm, Er, and Lu, produced increased locomotor activity across both light and dark phases, whereas other tested elements resulted in reduced activity relative to the control group Supplementary Figure S1. The statistical results for total distance traveled for all tested elements are provided in Supplementary Table S3.

### 3.3. Burst Count Movement of Zebrafish Following HM and REE Exposure

Burst movements reflect rapid, spontaneous swimming responses to external stimuli and are commonly associated with irregular locomotion and anxiety-like behavior under environmental stress. Consistent with total distance traveled, control larvae displayed higher burst frequencies during the dark phase than during the light phase, indicating normal light–dark modulation of locomotor reactivity.

Exposure to HMs and REEs significantly altered this baseline behavioral pattern in a phase-dependent manner. During the light phase, most compounds induced a pronounced hyperactive burst phenotype, with increased burst counts observed across HMs, LREEs, and HREEs Figure 4A, Supplementary Figure S2, suggesting elevated arousal or enhanced stimulus responsiveness. In contrast, a limited subset of elements, including Zn and several REEs, reduced burst activity relative to controls, indicating hypoactivity. Notably, Co consistently suppressed burst movements during the light cycle, reflecting a strong inhibitory effect on rapid acceleration behavior. During the dark phase, the behavioral response shifted toward a general suppression of burst activity, with most compounds decreasing burst counts compared with controls Figure 4B. Only a few elements maintained elevated burst activity in darkness, indicating compound-specific disruption of normal light–dark behavioral regulation. Overall, these results reveal distinct, light–dark-dependent burst activity patterns, highlighting burst behavior as a sensitive endpoint for detecting neurobehavioral toxicity. Detailed statistical analyses of burst activity during light, dark, and transition periods are summarized in Supplementary Table S4.

### 3.4. Swimming Orientation for Zebrafish Following HM and REE Exposure

In this study, the control larvae group exhibited a higher frequency of rotational movements during the dark phase, consistent with increased exploratory or anxiety-like behavior under low-light conditions. Exposure to HMs and REEs disrupted this baseline pattern in a compound- and phase-dependent pattern, revealing distinct rotational behavioral signatures. Overall, most REEs induced a sustained increase in rotation counts across both light and dark cycles, suggesting persistent alterations in sensorimotor coordination or heightened anxiety-like circling behavior. In contrast, several HMs, including Al, Sb, Cr, Mn, and Mo, suppressed rotational activity relative to controls, with Co, Mn, and Mo consistently reducing rotations across both phases Supplementary Figure S3.

During the light phase, most HMs and REEs significantly increased rotation counts compared with controls Figure 5A, indicating enhanced turning behavior and altered locomotor control under illuminated conditions. Notably, Co reduced rotational movements, while Zr produced no significant effect, highlighting element-specific differences. During the dark phase, increased rotation was primarily restricted to REE-treated groups, particularly La, Ce, Tb, Dy, and Tm, whereas most other treatments showed no deviation from controls Figure 5B. Detailed statistical analyses are summarized in Supplementary Table S5.

### 3.5. Measuring Different 2D Behavioral Endpoints of Zebrafish Larvae Using idtracker.ai

In this study, we evaluated zebrafish larvae across multiple 2D behavioral phenomics endpoints, including locomotor activity, exploratory behavior, movement orientation, and movement complexity, following exposure to selected HMs and REEs at 0.1 mg/L. Locomotor analysis revealed a predominantly suppressive behavioral profile, with several HMs and most REEs significantly reducing larval activity. These effects were characterized by decreased average swimming speed, reduced swimming and rapid movement durations, and increased freezing time Figure 6A–D, indicating impaired motor output and elevated stress responses. In contrast, the remaining metals did not produce statistically significant locomotor changes relative to controls. Exploratory behavior, assessed by center occupancy and thigmotaxis, was largely unaffected by most metals. Notably, Ni uniquely reduced time spent in the central area while increasing thigmotaxis, suggesting enhanced avoidance or anxiety-like behavior Figure 6E,F. Movement orientation analysis demonstrated that exposure to Zn and most REEs increased angular velocity, while a subset of HMs and REEs elevated meandering, reflecting disrupted directional control and unstable swimming trajectories Figure 6G,H. Other metals showed no measurable effects on orientation. Movement complexity, quantified using fractal dimension and entropy, was significantly reduced following exposure to Ni and multiple REEs, indicating simplified and less adaptive locomotor patterns. Decreased entropy in selected treatments further suggested irregular and unpredictable movement behavior Figure 6I,J. Complete statistical outcomes are provided in Supplementary Tables S6–S9.

### 3.6. Total Distance Chronology of Daphnia magna Following HM and REE Exposure

*Daphnia magna* exhibited higher locomotor activity during the light phase than during the dark phase, as reflected by the greater total distance traveled Supplementary Figure S4. Exposure to HMs and REEs altered total distance traveled in a compound- and phase-dependent manner.

HMs, including Sb, Cr, Co, Ga, Fe, and Mn, induced increased locomotor activity across both light and dark phases, whereas the remaining tested metals generally produced reduced activity relative to the control group Supplementary Figure S4. During the light phase, most HMs increased locomotor activity, whereas LREEs and HREEs generally reduced activity. Exposure to Ni, Pt, Sr, Zn, and Zr did not significantly alter locomotor activity compared with the control group Figure 7A.

During the dark phase, most tested HMs, except Ni, Pt, V, and Zr, significantly increased total distance traveled compared with the control group. In contrast, all tested REEs significantly reduced locomotor activity during this phase Figure 7B. Detailed statistical results for total distance traveled during the light, dark, and transition periods are provided in Supplementary Table S10.

### 3.7. Burst Count of Daphnia magna Following HM and REE Exposure

Following exposure to HMs and REEs, *Daphnia magna* exhibited an overall suppression of burst movements, as indicated by reduced burst counts across most treatments (Supplementary Figure S5). This response contrasts sharply with that of zebrafish larvae, which frequently displayed elevated burst activity, highlighting pronounced species-specific differences in rapid locomotor behavior. During the light phase, only a limited number of HMs, particularly iron and Li, induced increased burst activity, whereas most other HMs and all REEs significantly reduced burst counts relative to controls Figure 8A. In the dark phase, a similar pattern persisted, with Fe remaining the only HM that consistently increased burst activity. In contrast, the majority of HMs and REEs suppressed burst movements, while Li, Mn, and Eu showed no significant deviations from control levels Figure 8B. These findings demonstrate that *Daphnia magna* responds to HMs and REEs with a predominantly hypoactive burst phenotype, particularly under dark conditions. Detailed statistical outcomes for burst activity during light, dark, and transition periods are provided in Supplementary Table S11.

### 3.8. Swimming Orientation of Daphnia magna Following HM and REE Exposure

In *Daphnia magna*, exposure to HMs and REEs resulted in a different rotation count compared with that of zebrafish larvae. Our results indicated that several HMs, including Sb, Mn, Mo, Ni, and Zn, consistently increased rotation counts during both light and dark cycles. In contrast, REEs exhibited uniformly low rotation counts, with values remaining close to zero and showing minimal variability, indicating little effect on rotational behavior. This suggests that exposure to these REEs produced negligible changes in rotation count relative to the control group Supplementary Figure S6. During the light phase, several HMs, including Al, Sb, Fe, Li, Pt, Sr, Sn, and Zn, significantly increased rotation counts relative to controls, indicating altered locomotor turning behavior. In contrast, Mn, and Mo exposure reduced rotational activity, while the remaining HMs did not induce significant changes. REE-treated groups exhibited negligible variation in rotation counts during the light period Figure 9A. During the dark phase, REEs likewise showed no significant effects on rotational behavior. In contrast, a broader range of HMs, including Al, Sb, Bi, Cr, Fe, Ni, Pt, Sr, Sn, and Zn, elicited increased rotational movements compared with controls, indicating enhanced turning behavior under dark conditions Figure 9B. Detailed statistical analyses of rotation count during light, dark, and transition periods are summarized in Supplementary Table S12.

### 3.9. Measuring Different 2D Behavioral Endpoints of Daphnia magna Using idtracker.ai

Unlike zebrafish larvae, *Daphnia magna* exhibited a distinct and predominantly suppressive behavioral profile across multiple 2D phenomics endpoints following exposure to HMs and REEs at 0.1 mg/L. Locomotor activity, exploratory behavior, movement orientation, and movement complexity were analyzed to characterize phenotypic responses and enable direct comparison with zebrafish larvae. Locomotor analysis revealed that only a limited number of elements induced significant effects in *Daphnia magna*. Among HMs, Sr reduced average swimming speed and swimming duration while increasing freezing behavior. Among REEs, Eu, Tm, and Y similarly suppressed swimming activity and increased freezing time. In addition, Mn and Zn selectively reduced rapid movement duration, whereas all other tested elements produced no statistically significant locomotor activity changes relative to controls Figure 10A–D. Overall, far fewer metals elicited measurable locomotor effects in *Daphnia magna* compared with zebrafish larvae, indicating a narrower behavioral response range. Exploratory behavior, assessed by center occupancy and thigmotaxis, remained unchanged across all exposure groups, suggesting minimal effects on exploration-related behavior Figure 10E,F. Likewise, movement orientation parameters, including angular velocity and meandering, showed no significant differences from controls, indicating preserved directional control Figure 10G,H. Measures of movement complexity, including fractal dimension and entropy, were also unaffected, in contrast to the reduced complexity observed in *Daphnia magna* Figure 10I,J. Collectively, these results demonstrate that *Daphnia magna* neonates display a more limited and primarily suppressive phenomics response to HMs and REEs. Detailed quantitative data are provided in Supplementary Tables S13–S16.

### 3.10. PCA and Clustering of Phenomics Data

Clustering is a statistical technique that categorizes data points based on their similarity, ensuring that items within the same cluster are more similar to one another than those in separate clusters. Our clustering results demonstrate that multivariate behavioral phenomics effectively differentiates between toxicity patterns of HMs and REEs in zebrafish larvae and *Daphnia magna*. In zebrafish larvae, the first two principal components explained 63.1% of the total variance, indicating that metal-induced behavioral alterations were structured and non-random. Several REEs, such as Ho, Dy, Eu, Tu, and Yb, clustered closely with highly disruptive HMs, reflecting shared behavioral signatures characterized by locomotor suppression, increased freezing, altered turning behavior, and reduced movement complexity. Similar clustering of chemically distinct compounds has been reported in zebrafish behavioral phenomics as an indicator of convergent neurotoxic outcomes rather than shared chemical structure [68,69].

In contrast, PCA of *Daphnia magna* data explained 57.3% of the variance and revealed tighter clustering for most REEs near control conditions, indicating weaker or less multidimensional behavioral effects. Heatmap-based hierarchical clustering further showed that toxicity patterns in *Daphnia magna* were driven primarily by changes in basic locomotor endpoints, such as total distance and burst activity, rather than higher-order behavioral metrics. This reduced phenotypic dimensionality has been observed in invertebrate behavioral toxicology and reflects fundamental differences in neural organization and sensory processing compared with vertebrates. Collectively, PCA and heatmap clustering confirms that behavioral toxicity profiles are both compound-specific and species-dependent, reinforcing the necessity of multi-species phenomics approaches for robust ecological risk assessment.

### 3.11. Toxicity Ranking and Cross-Species Sensitivity Comparison

The toxicity-ranking index integrates all assessed behavioral endpoints, such as locomotor activity, exploratory behavior, and orientation-related movements Figure 11, into a single composite score. This score reflects the overall neurobehavioral impact of each HMs and REEs. Higher values indicate greater deviation from control behavior, indicating increased toxicity and enabling direct comparison of metal effects between zebrafish larvae and *Daphnia magna.* Individual rank scores for each element are provided in the Supplementary Tables S17 and S18. The findings clearly highlight the differences between the two species’ responses. Many HMs and REEs that significantly disrupted zebrafish larvae’s behavior had a lesser effect on *Daphnia magna*, whereas others exhibited the opposite pattern. For example, HMs such as Ce can be highly toxic to larvae but have weaker effects on *Daphnia magna*, whereas Mo is more toxic to *Daphnia magna*. These variations likely stem from differences in species-specific physiology, nervous system structure, and metal absorption and distribution within their bodies.

To compare the sensitivity patterns between the two models, we used Spearman’s rank correlation. The low correlation observed (Spearman’s r = −0.0337, *p* = 0.8521; 95% CI: −0.3818 to 0.32) Figure 12 indicates that toxicity profiles differ across species, providing quantitative support for multi-species evaluation in neurotoxic risk assessment. This suggests that the toxicity pattern in zebrafish does not closely match that in *Daphnia magna*. This underscores the need to use multiple aquatic models to assess metal-induced neurobehavioral effects. Overall, the integrated ranking approach indicates that HMs and REEs elicit distinct behavioral changes across species. These findings underscore the importance of multi-species testing to gain a more comprehensive and realistic understanding of ecological risks.

## 4. Discussion

### 4.1. Phenomics Detection of Neurobehavioral Toxicity Induced by HMs and REEs in Aquatic Models

This study offers a detailed comparison of the toxic impacts of HMs and REEs on locomotor behavior in two commonly used aquatic models suitable for high-throughput toxicity assessment: zebrafish larvae [56] and *Daphnia magna* neonates [70]. This study employs a phenomics approach with multiple behavioral endpoints, building on the well-established practice of cross-species comparisons in ecotoxicology [24,71]. It uses both fish and water flea models, which have been shown to have generally similar sensitivities in previous research [8,72]. While most research has concentrated on mortality rates and developmental abnormalities in these species, fewer studies have explored how multiple toxicants impact locomotor activity, particularly in pesticide exposure [8,72]. Our principal component analysis and hierarchical clustering identified clear species-specific behavioral response patterns. Zebrafish larvae exhibited more uniform and reliable responses, whereas *Daphnia magna* showed greater variability and dispersion. These differences likely stem from fundamental physiological, neurodevelopmental, and stimulus-response variations among species, influencing both the direction and strength of behavioral changes following exposure to HMs and REEs.

### 4.2. Multidimensional Behavioral Profiling Uncovers Species-Specific Patterns of Locomotor Toxicity

Living organisms display many complex behaviors that are difficult to measure and compare across species. In this study, we used a high-throughput behavioral tracking platform combined with an omics-style analytical approach to perform multidimensional assessments of locomotor responses in zebrafish larvae and *Daphnia magna* exposed to HMs and REEs. Clustering methods are frequently used to analyze multivariate datasets in areas such as transcriptomic and gene expression profiling [73,74]. They have also been effectively applied to high-dimensional behavioral datasets, as shown in prior zebrafish studies, for classifying neuroactive compounds with similar mechanisms of action. Building on this concept, we applied an omics-based analytical approach to evaluate 13 locomotor parameters. These included total distance traveled, burst and rotation counts, mean velocity, angular velocity, meandering, swimming ratio, thigmotaxis, central area occupancy, rapid movement duration, fractal dimension, entropy, and freezing time in two aquatic model organisms exposed to metals and REEs. Even at sub-toxic levels, these substances induced noticeable behavioral changes, appearing as either hyperactivity or hypoactivity in both species. However, assessing toxicity solely through locomotor parameters proved challenging, as responses often differed between zebrafish and *Daphnia magna*. For instance, certain compounds caused complete immobilization in one species, while inducing only altered swimming patterns in the other [8]. These findings suggest that HMs and REEs may operate via species-specific toxicological pathways, probably shaped by variations in neurophysiology and the structural diversity and mechanisms of the chemicals [75].

### 4.3. Comparative Sensitivity of Zebrafish Larvae and Daphnia magna to HMs and REEs

Analysis of locomotor endpoints revealed that HMs typically induce more severe toxic effects than REEs in both zebrafish and *Daphnia magna*. This trend is consistent with earlier research showing that HMs tend to cause greater behavioral disruptions due to their higher bioavailability and well-established neurotoxic mechanisms [76,77]. In contrast, while REEs could induce measurable behavioral changes, these effects were generally less intense and less consistent across endpoints, indicating a comparatively minor immediate impact on locomotor performance [78]. Our cross-species assessment revealed both similar and distinct response patterns. Although significant behavioral changes were observed in both models following toxicant exposure, their responses differed: zebrafish larvae tended to become hyperactive under HM exposure. In contrast, *Daphnia magna* neonates more often showed hypoactivity. These variations likely stem from fundamental differences in neurophysiology and ecological adaptations. Zebrafish, being vertebrates, possess more complex nervous systems and exhibit stronger visual startle responses, especially in dark conditions. In contrast, *Daphnia magna* activity usually peaks in light, reflecting differences in circadian and sensory adaptations [8]. These species-specific locomotor patterns highlight the importance of using multiple model organisms in ecotoxicological assessments. Because LC_50_ values varied considerably among the tested HMs and REEs, the common behavioral exposure concentration of 0.1 mg/L represented different fractions of the corresponding LC_50_ values. Therefore, the behavioral responses observed at this concentration should be interpreted as sublethal effects under a standardized common exposure concentration rather than as LC_50_-normalized measures of the intrinsic toxicity of individual elements. The use of a concentration of 0.1 mg/L enabled direct comparison of behavioral disruption across the tested elements and between zebrafish larvae and *Daphnia magna* under non-lethal experimental conditions. Accordingly, the LC_50_ and behavioral phenomics results should be considered complementary endpoints, representing acute lethal toxicity and sublethal behavioral effects, respectively.

### 4.4. Differential Locomotor Responses Indicate Distinct Toxicological Pathways for HMs and REEs

The diverse locomotor phenotypes observed after exposure to HMs and REEs suggest that these metals likely act through distinct toxicological pathways. HMs consistently induced more severe and coherent behavioral disruptions in both zebrafish larvae and *Daphnia magna* neonates. This aligns with their known capacity to disrupt ion homeostasis, neurotransmission, and mitochondrial function via mechanisms including calcium channel dysregulation, oxidative stress, and acetylcholinesterase inhibition [76,79,80]. In contrast, REEs induced zebrafish larvae and *Daphnia magna* to move in less predictable, more variable patterns. This indicates they may operate through different, potentially milder mechanisms, such as interfering with calcium-dependent signaling pathways, altering membrane permeability, or indirectly disrupting metabolism, rather than causing direct neurotoxicity [75,81,82]. The different species exhibit distinct behaviors, supporting the idea that there are species-specific ways for them to take in, eliminate, and respond to toxins in their nervous systems [24]. These differences affect how sublethal toxicity affects their functions. Notably, varied locomotor responses were observed at the standardized, sublethal screening concentrations, highlighting the importance of multi-endpoint behavioral phenomics in differentiating toxicant types that may appear similar when assessed solely by mortality metrics. Overall, these results demonstrate that analyzing a range of locomotor traits across species provides essential insights into the distinct toxicity pathways of HMs and rare earth elements, enhancing mechanistic understanding and ecological significance in aquatic risk evaluations.

### 4.5. Implications of Dual-Species Phenomics Screening for Environmental Risk Assessment

This study highlights the critical value of a dual-species behavioral phenomics framework for enhancing the sensitivity and ecological relevance of aquatic environmental risk assessment. Zebrafish larvae and *Daphnia magna* are well-established, high-throughput aquatic models that can be evaluated using comparable exposure designs, concentration ranges, and automated behavioral tracking platforms, enabling efficient parallel screening under standardized protocols Figure 1. Such methodological alignment allows for the rapid assessment of large chemical libraries while facilitating direct cross-species comparisons of toxicity profiles [7,69]. To the best of our knowledge, this work represents the first large-scale comparative phenomics assessment that extends beyond conventional acute toxicity endpoints to systematically characterize locomotor and neurobehavioral toxicity induced by HMs and REEs across multiple behavioral dimensions. Despite their experimental compatibility, the two models exhibited fundamentally distinct behavioral toxicity signatures. Zebrafish larvae displayed multidimensional neurobehavioral disturbances involving locomotion, orientation, exploratory behavior, and movement complexity, reflecting the heightened sensitivity of vertebrate neural circuits to sublethal toxic stress [83]. In contrast, *Daphnia magna* responses were primarily characterized by alterations in basal locomotor activity and burst dynamics, which are well-recognized indicators of physiological stress and energetic imbalance in invertebrates [18,84]. Multivariate analyses, including principal component analysis and hierarchical clustering Figure 11, together with species correlation analyses based on behavioral numeric differences Figure 12; Supplementary Tables S17 and S18, revealed limited concordance in toxicity rankings between species, highlighting the influence of species-specific neurophysiology, sensory processing, and metal uptake pathways. Collectively, this dual-model framework has the potential to improve environmental hazard assessments for both established contaminants, such as HMs, and emerging pollutants, such as REEs. Future studies should build on this by integrating molecular and metabolic profiling, thereby enabling a deeper understanding of toxicity mechanisms and strengthening the evidence base for regulatory decisions.

## 5. Conclusions

In this study, we performed a large-scale, dual-species behavioral phenomics assessment to compare the neurobehavioral toxicity of 17 HMs and 16 REEs in zebrafish larvae and *Daphnia magna* neonates. By integrating LC_50_-based acute toxicity with 13 high-resolution locomotor and exploratory endpoints measured at a uniform sublethal concentration (0.1 mg/L), we revealed toxicity patterns that were not evident from mortality data alone. Several REEs, including Dy, Ho, Eu, Tm, and Yb, induced pronounced behavioral alterations in zebrafish larvae, such as disrupted photomotor responses, altered burst and rotation activity, reduced movement complexity, and increased freezing behavior, with effect magnitudes comparable to or exceeding those of multiple HMs.

Zebrafish larvae exhibited broad and consistent behavioral sensitivity across locomotor, orientation, and complexity endpoints, whereas *Daphnia magna* displayed a narrower and predominantly suppressive behavioral response, with significant effects observed for only a subset of metals. Multivariate analyses (PCA and hierarchical clustering) clearly separated metals by species-specific behavioral signatures, and composite toxicity ranking showed no significant correlation between the two models (Spearman’s r = −0.0337, *p* = 0.8521; 95% CI: −0.3818 to 0.32), Figure 12, indicating limited concordance in their behavioral toxicity rankings and supporting species-specific differences in sensitivity profiles. Notably, behavioral toxicity rankings did not align with LC_50_ values, demonstrating that sublethal neurobehavioral disruption occurs independently of acute lethality. Collectively, these results establish multi-endpoint behavioral phenomics as a sensitive and discriminative approach for metal risk assessment and underscore the ecological relevance of incorporating both vertebrate and invertebrate models when evaluating HM and REE contamination.

## Figures and Tables

**Figure 1:**
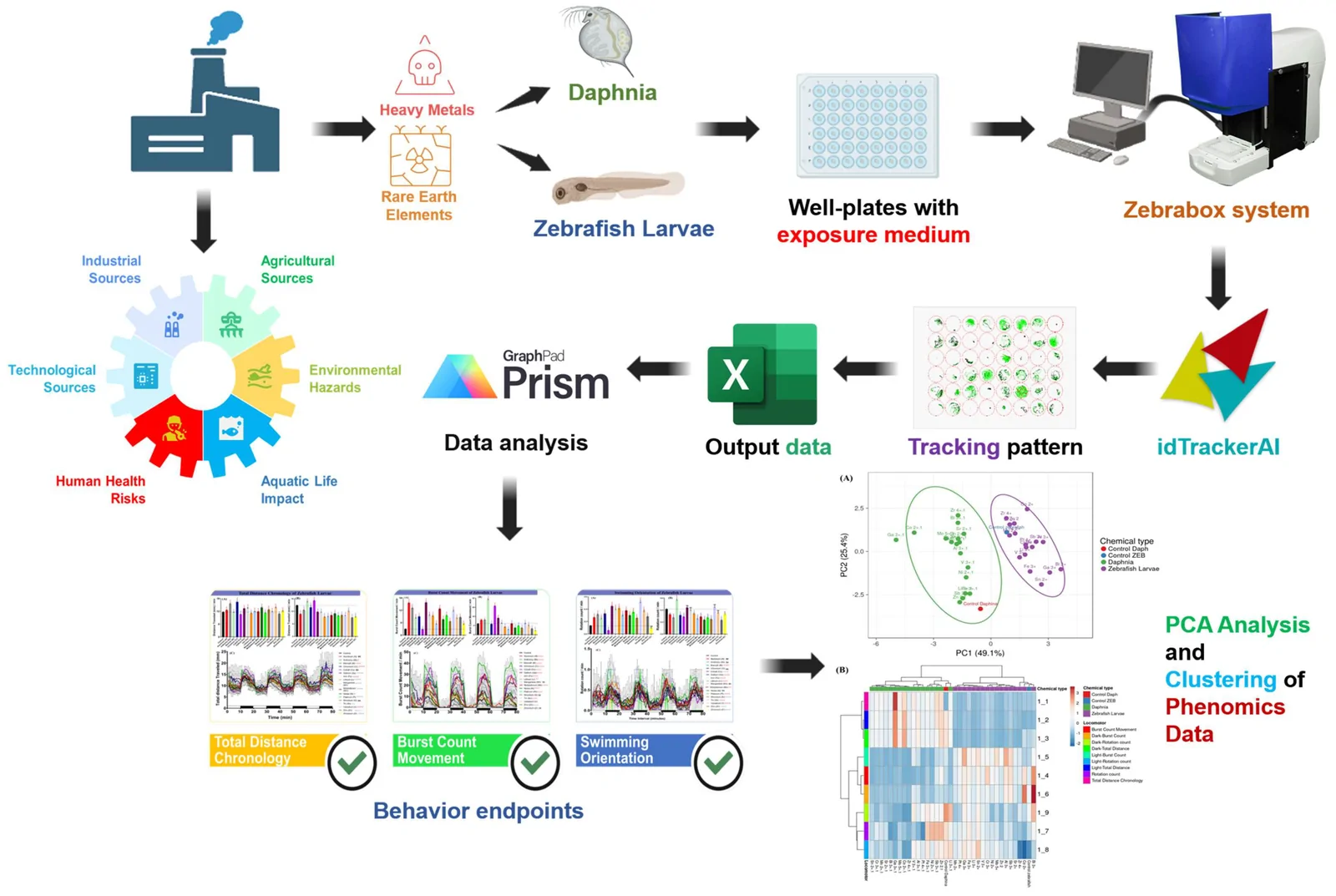
Schematic overview of the experimental workflow for assessing behavioral toxicity of HMs and REEs in aquatic model organisms. Industrial, agricultural, technological, and environmental sources release contaminants that impact aquatic life and human health. In this workflow, daphnia and zebrafish larvae are exposed in multi-well plates, and locomotor activity is recorded using the Zebrabox system. Trajectories are tracked with IdTracker.ai, processed in Excel, and analyzed in GraphPad Prism. Key behavioral endpoints (total distance, burst count, swimming orientation) are quantified. Multivariate phenomic data are further explored via (**A**) PCA and (**B**) hierarchical clustering to discriminate chemical exposure effects.

**Figure 2:**
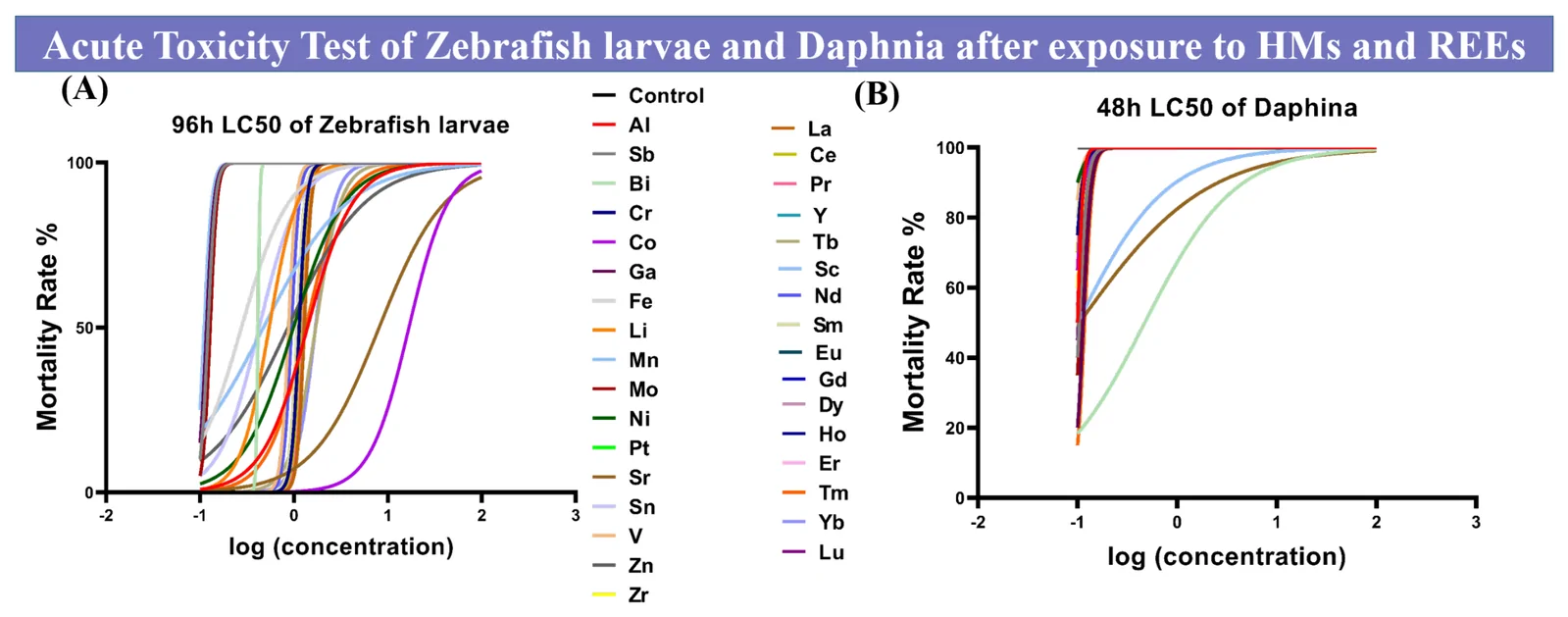
Acute toxicity dose–response curves of zebrafish larvae and *Daphnia magna* following exposure to HMs and REEs. (**A**) 96 h LC_50_ of zebrafish larvae. (**B**) 48 h LC_50_ of *Daphnia magna*. Left panel shows concentration–response relationships for the zebrafish chemical set; right panel shows the corresponding curves for the *Daphnia magna* test. Each colored line represents an individual HM or REE, illustrating differential potency and slope. Curves were generated from standardized acute toxicity assays measuring mortality or immobilization endpoints. The sigmoidal profiles enable the calculation of LC_50_ values and allow for direct comparison of relative toxicities across the tested HMs and REEs in both aquatic model species.

**Figure 3:**
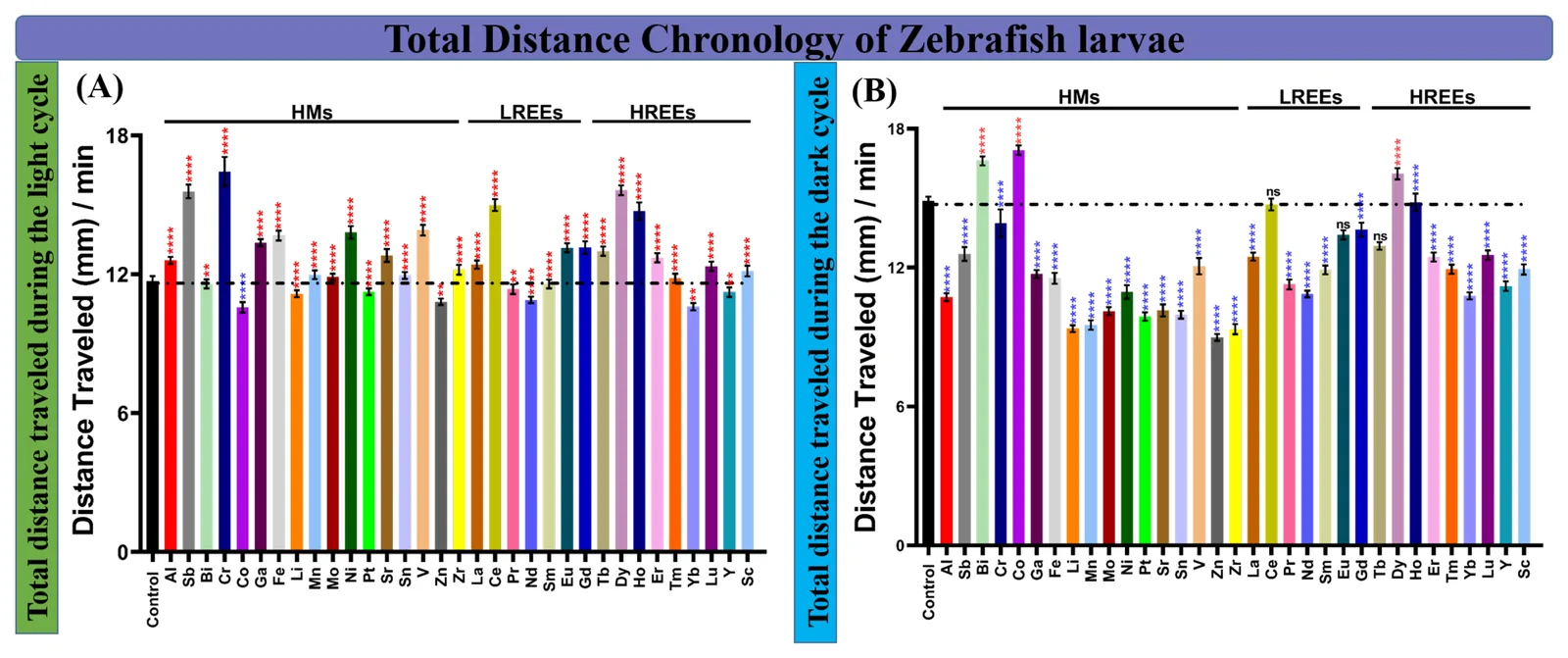
Average distances traveled by zebrafish larvae in the control group and groups exposed to 0.1 mg/L HMs and REEs for 24 h. (**A**) Distance traveled during the light phase. (**B**) Total distance covered during the dark cycle. Data are presented as mean ± SEM. Differences between light and dark conditions relative to the control group were analyzed using one-way ANOVA followed by Dunn’s multiple comparisons test. Statistical significances are denoted as follows: ns (not significant), ** *p* < 0.01, and **** *p* < 0.0001 (*n* = 48).

**Figure 4:**
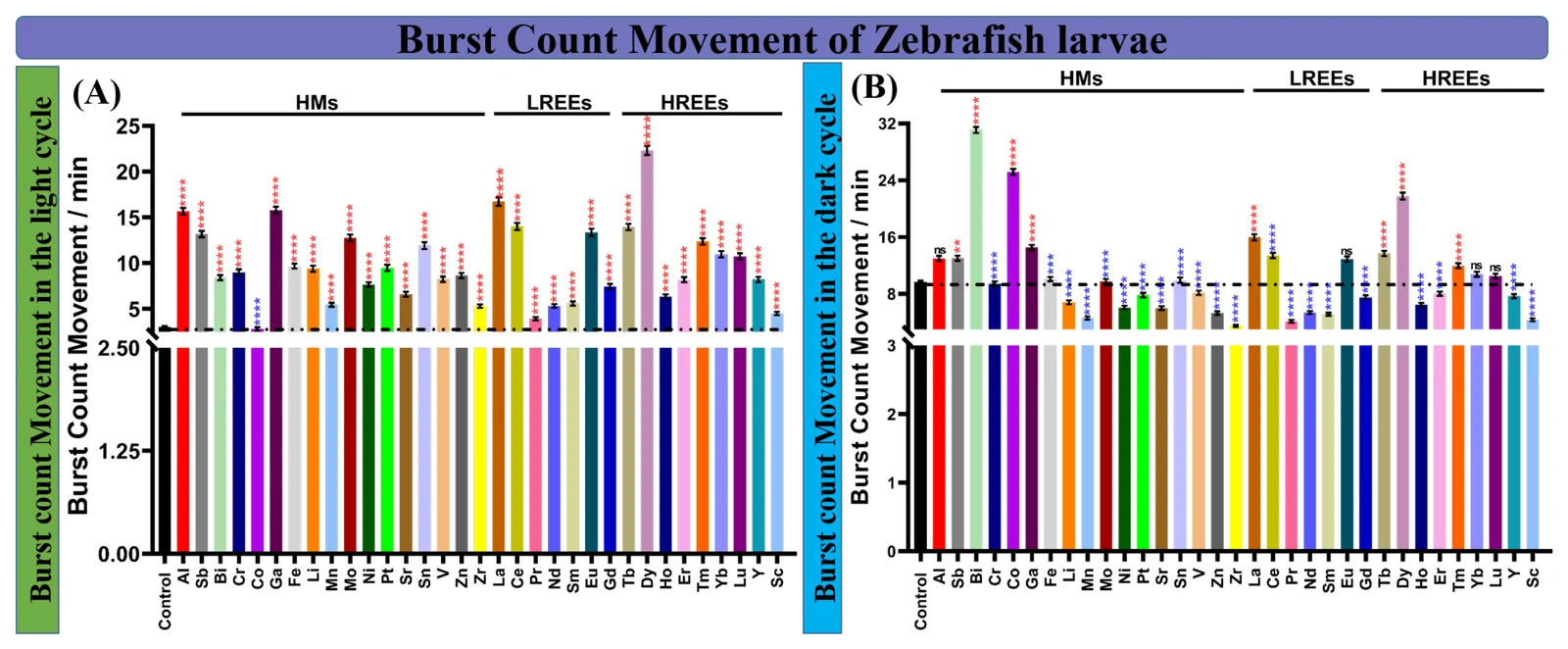
Total burst counts in zebrafish larvae exposed to control and 0.1 mg/L HMs and REEs for 24 h. (**A**) Burst counts recorded during the light phase. (**B**) Burst counts recorded during the dark phase. Data are presented as mean ± SEM. Differences between light and dark conditions relative to the control group were analyzed using one-way ANOVA followed by Dunn’s multiple comparisons test. Statistical significances are denoted as follows: ns (not significant), ** *p* < 0.01, *** *p* < 0.001, and **** *p* < 0.0001 (*n* = 48).

**Figure 5:**
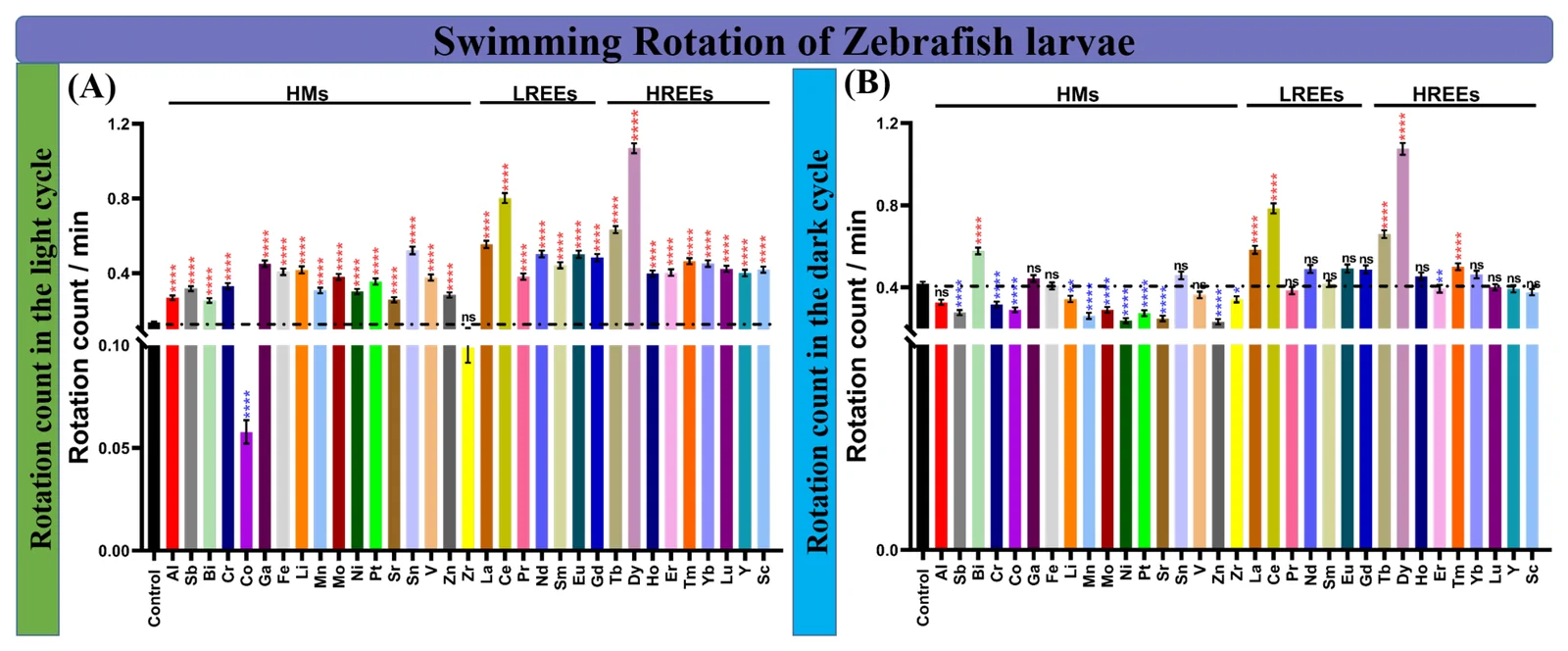
Total rotation counts in zebrafish larvae exposed to control conditions or 0.1 mg/L HMs and REEs for 24 h. (**A**) Rotations during the light phase. (**B**) Rotations during the dark phase. Data are presented as mean ± SEM. Differences between light and dark conditions relative to the control group were analyzed using one-way ANOVA followed by Dunn’s multiple comparisons test. Statistical significances are denoted as follows: ns (not significant), * *p* < 0.05, ** *p* < 0.01, and **** *p* < 0.0001 (*n* = 48).

**Figure 6:**
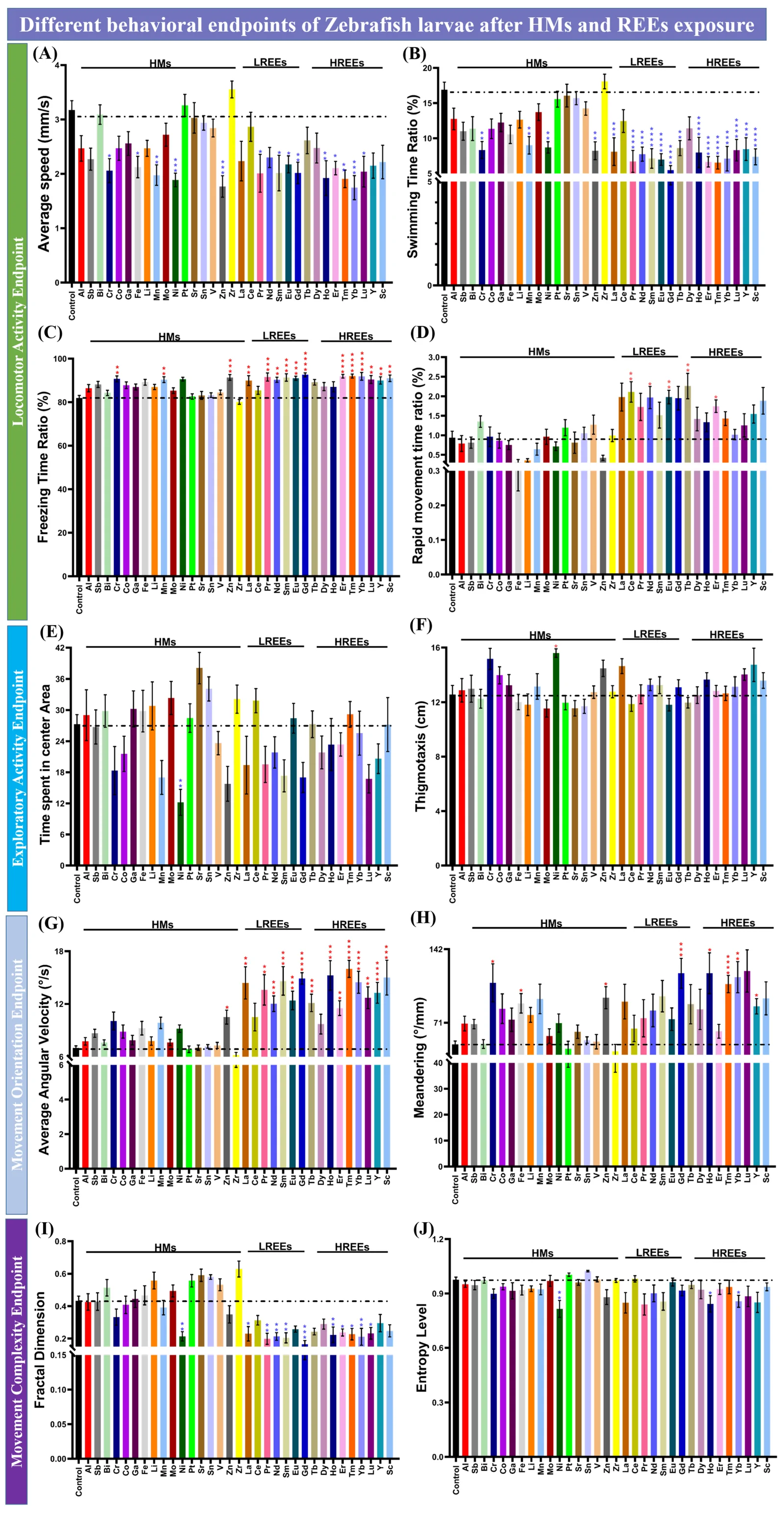
Larvae are exposed to different HMs and REEs at 0.1 mg/L for 24 h. Ten behavioral endpoints were measured: (**A**) average speed, (**B**) freezing time, (**C**) swimming time, (**D**) rapid movement, (**E**) time spent in the center, (**F**) thigmotaxis, (**G**) average angular velocity, (**H**) meandering, (**I**) fractal dimension and (**J**) entropy. Data are presented as mean ± SEM and were analyzed using one-way ANOVA followed by Dunn’s multiple comparisons test. Sample size: *n* = 18. Statistical significances are denoted as follows: ns (not significant), * *p* < 0.05, ** *p* < 0.01, *** *p* < 0.001, and **** *p* < 0.0001.

**Figure 7:**
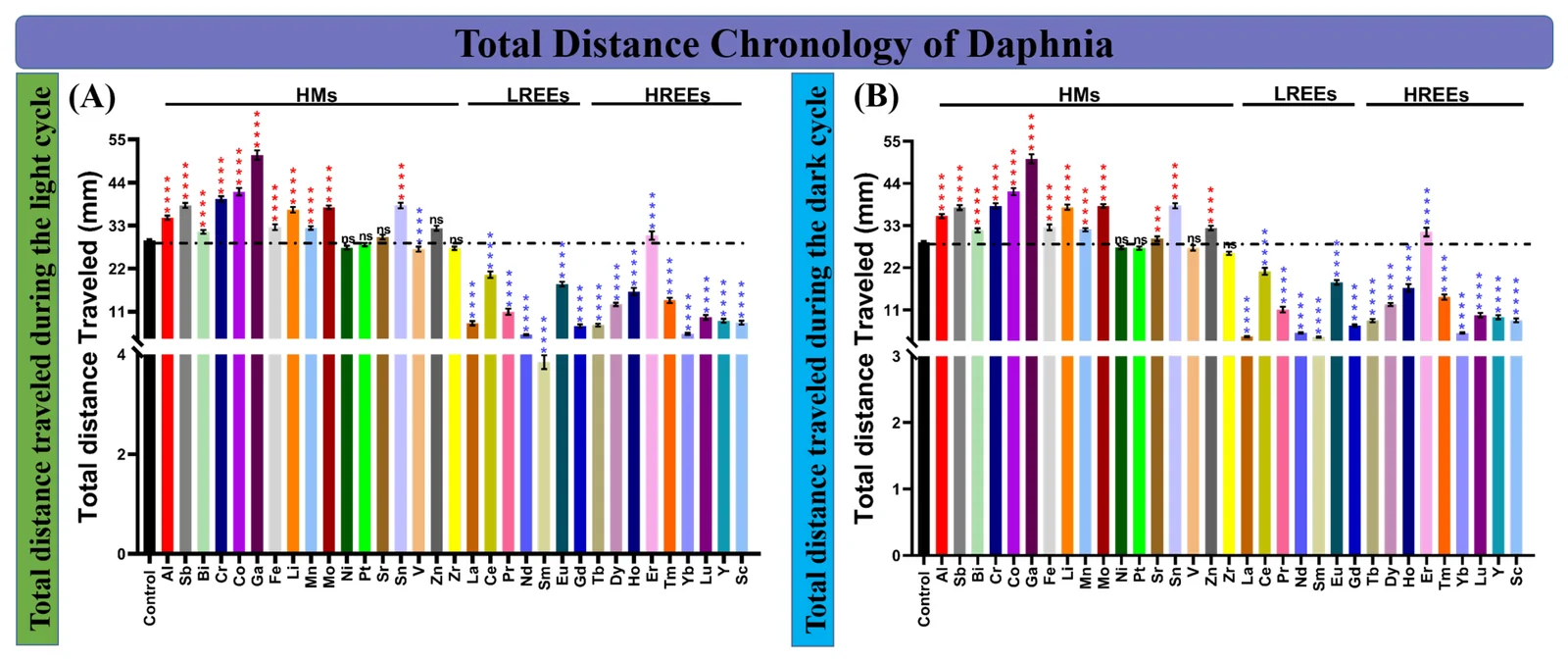
Average distances traveled by *Daphnia magna* in the control group and groups exposed to 0.1 mg/L HMs and REEs or 24 h are shown. (**A**) Distance traveled during the light phase. (**B**) Total distance covered during the dark cycle. Data are presented as mean ± SEM. Differences between light and dark conditions relative to the control group were analyzed using one-way ANOVA followed by Dunn’s multiple comparisons test. Statistical significances are denoted as follows: ns (not significant), *** *p* < 0.001, and **** *p* < 0.0001 (*n* = 48).

**Figure 8:**
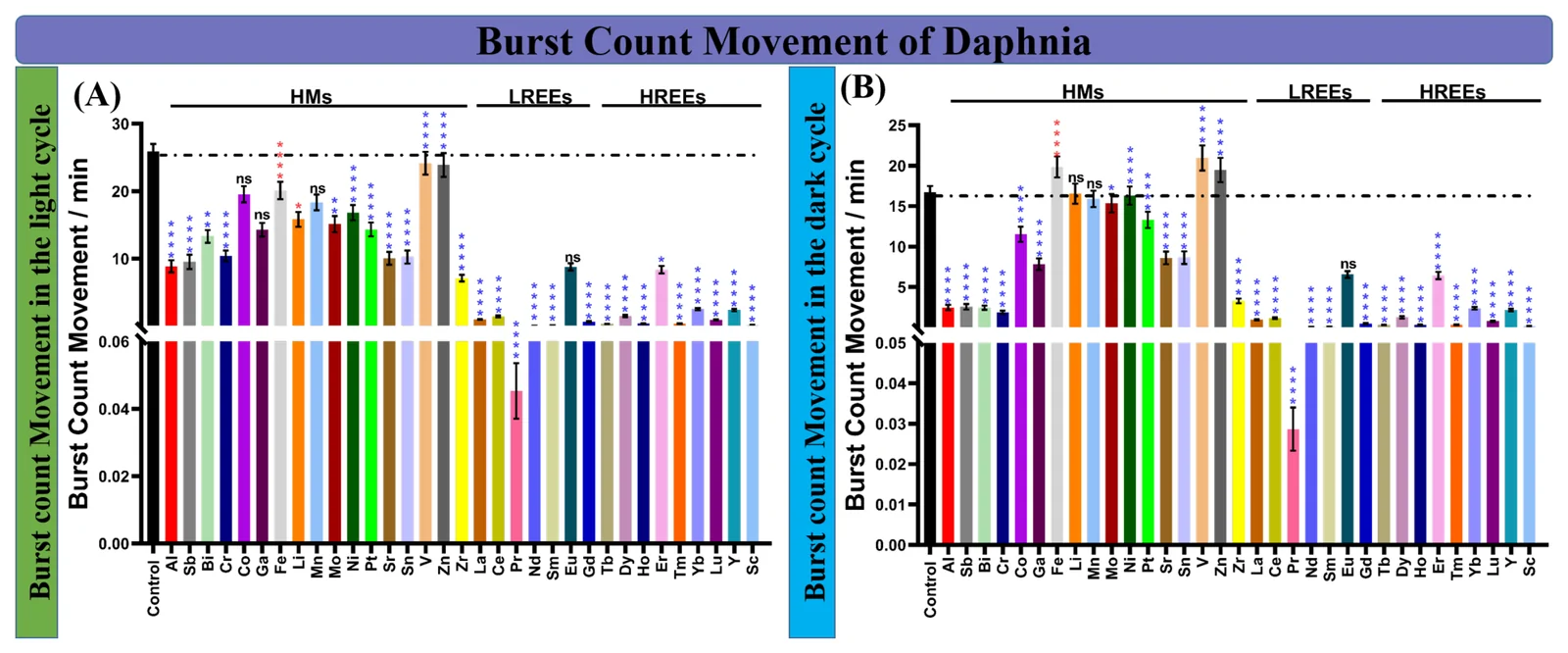
Total burst counts in *Daphnia magna* exposed to control and 0.1 mg/L HMs and REEs for 24 h. (**A**) Burst counts recorded during the light phase. (**B**) Burst counts recorded during the dark phase. Data are presented as mean ± SEM. Differences between light and dark conditions relative to the control group were analyzed using one-way ANOVA followed by Dunn’s multiple comparisons test. Statistical significances are denoted as follows: ns (not significant), * *p* < 0.05, ** *p* < 0.01, and **** *p* < 0.0001 (*n* = 48).

**Figure 9:**
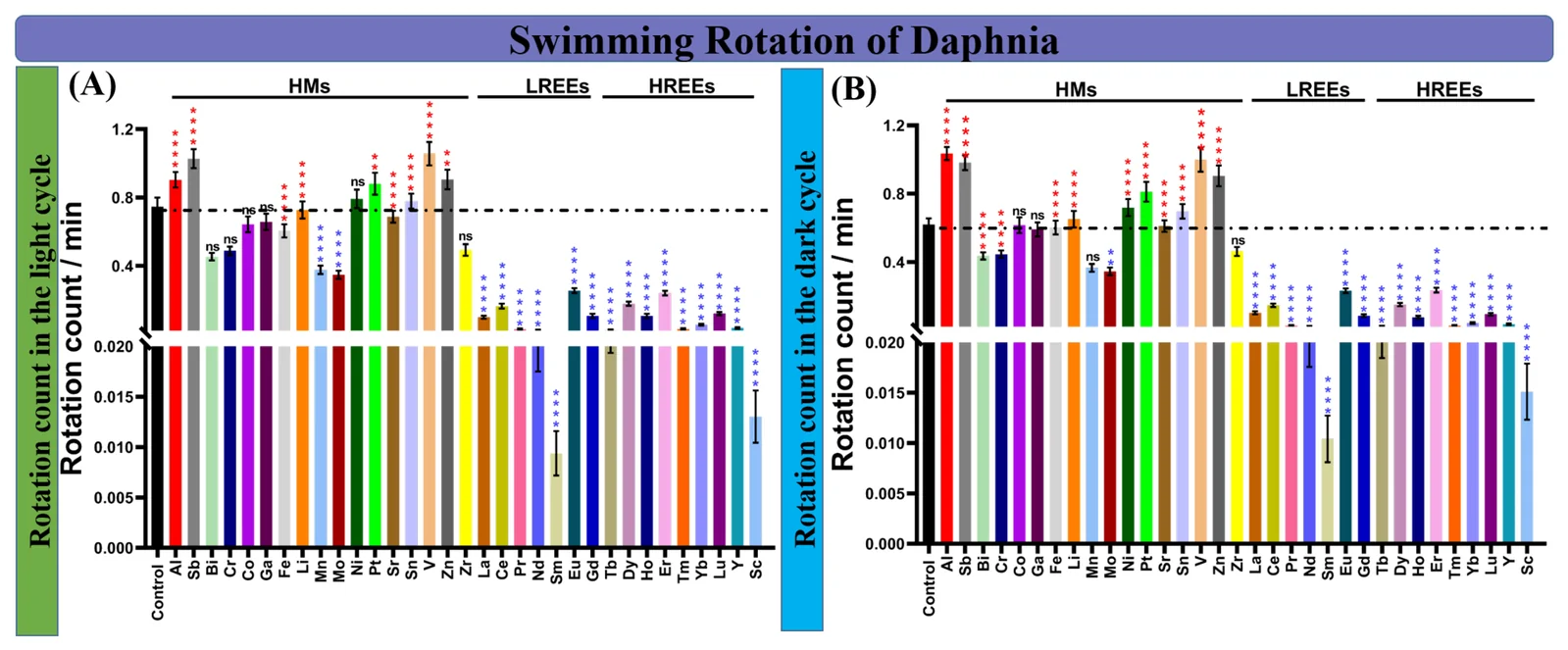
Total rotation counts in *Daphnia magna* exposed to control conditions or 0.1 mg/L HMs and REEs for 24 h. (**A**) Rotations during the light phase. (**B**) Rotations during the dark phase. Data are presented as mean ± SEM. Differences between light and dark conditions relative to the control group were analyzed using one-way ANOVA followed by Dunn’s multiple comparisons test. Statistical significances are denoted as follows: ns (not significant), * *p* < 0.05, ** *p* < 0.01, and **** *p* < 0.0001 (*n* = 48).

**Figure 10:**
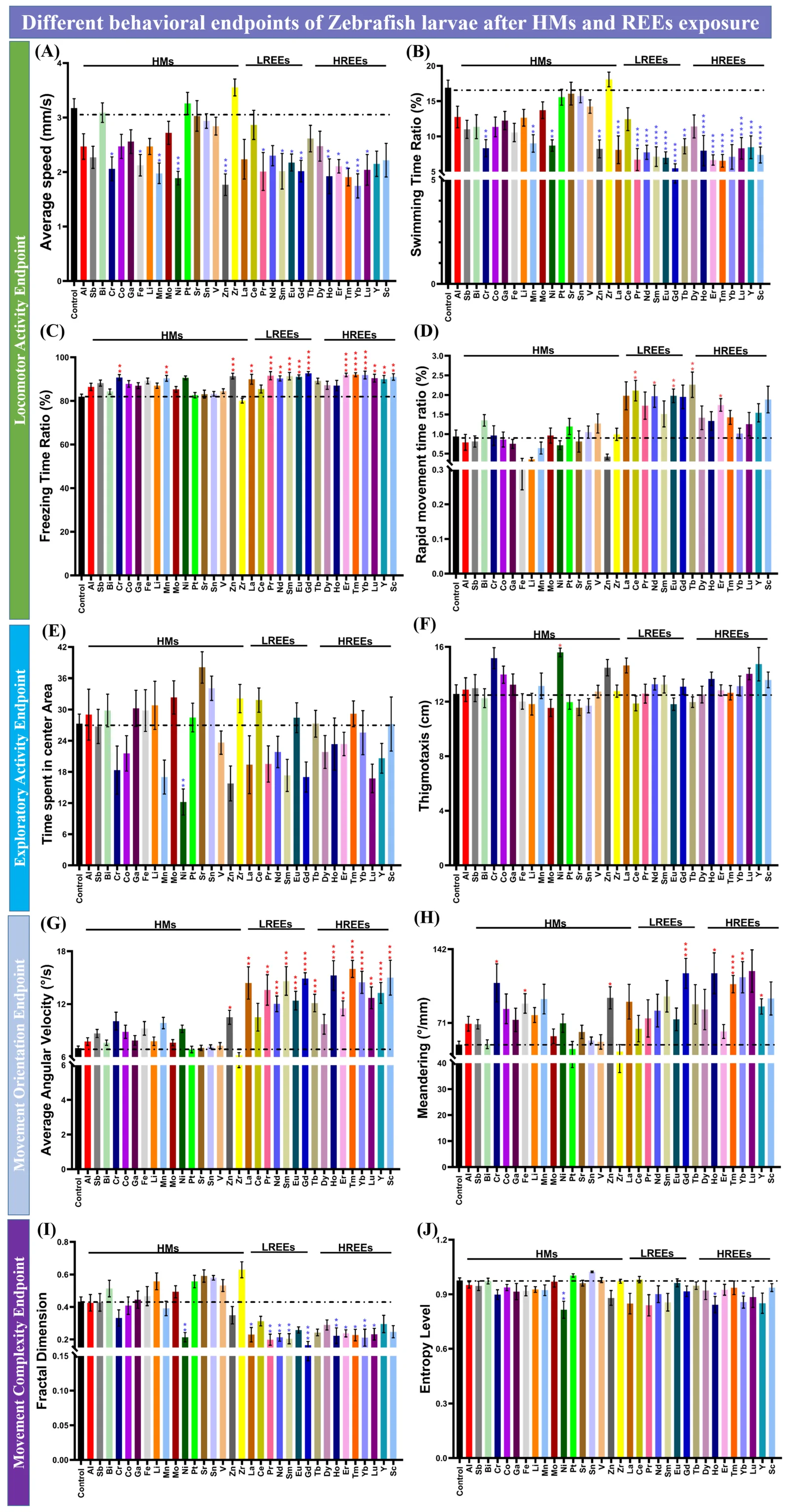
*Daphnia magna* are exposed to different HMs and REEs at 0.1 mg/L for 24 h. Ten behavioral endpoints were measured: (**A**) average speed, (**B**) freezing time, (**C**) swimming time, (**D**) rapid movement, (**E**) time spent in the center, (**F**) thigmotaxis, (**G**) average angular velocity, (**H**) meandering, (**I**) fractal dimension, and (**J**) entropy. Data are presented as mean ± SEM and were analyzed using one-way ANOVA followed by Dunn’s multiple comparisons test. Sample size: *n* = 18. Statistical significances are denoted as follows: ns (not significant), * *p* < 0.05, ** *p* < 0.01, *** *p* < 0.001, and **** *p* < 0.0001.

**Figure 11:**
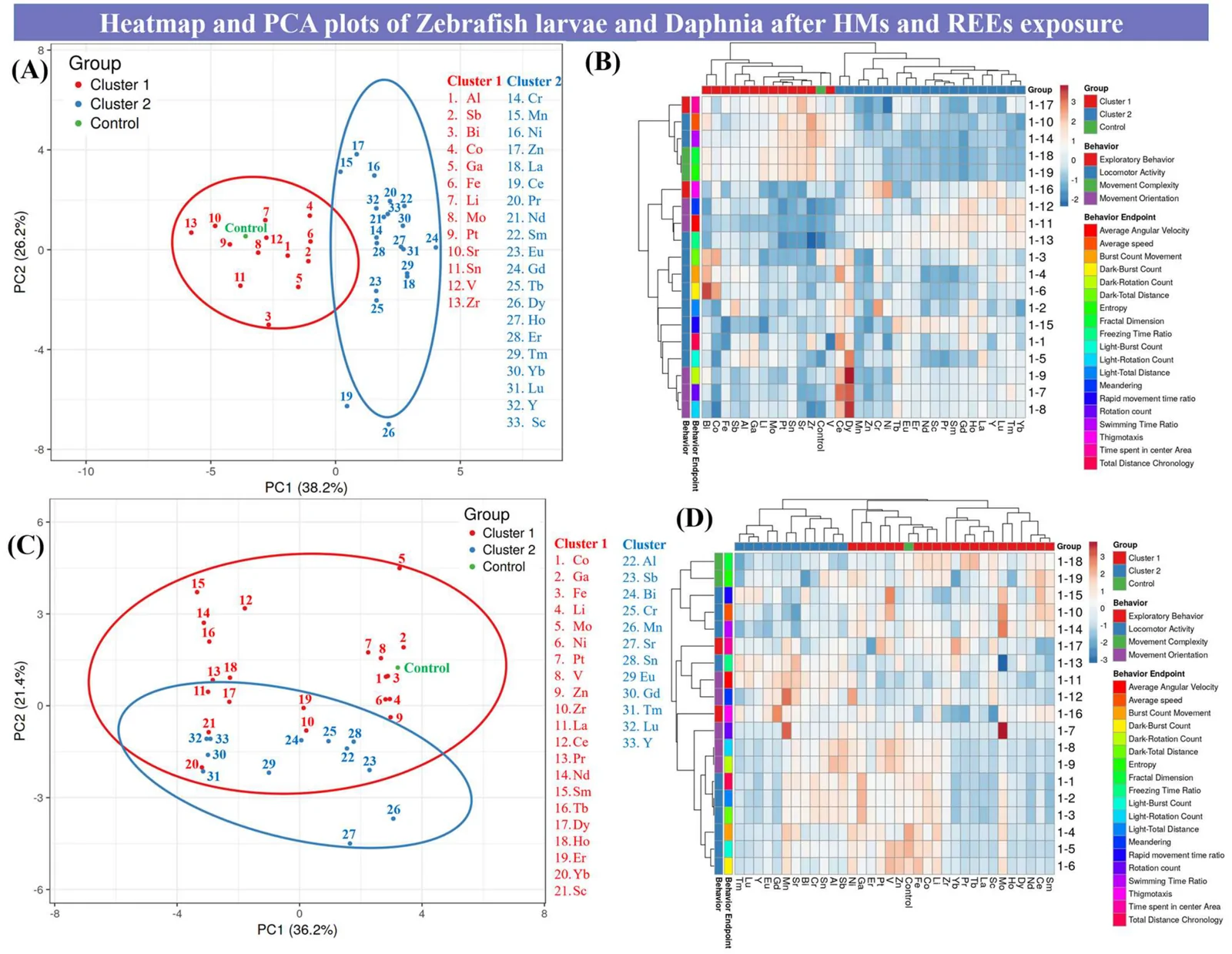
Hierarchical clustering of locomotor activity changes in zebrafish larvae and *Daphnia magna* based on HM and REE exposure. Principal component analysis (PCA) plots (**A**,**C**) and corresponding heatmaps (**B**,**D**) illustrate the behavioral effects of 17 HMs and 16 REEs in zebrafish larvae (*Danio rerio*) and *Daphnia magna* neonates. PCA was applied to multiple locomotor and behavioral endpoints to reduce data dimensionality and visualize clustering patterns. In zebrafish larvae, the first two principal components explain 63.1% of the total variance, while in *Daphnia magna* they account for 57.3%, indicating that the two-dimensional projections capture the majority of metal-induced behavioral variation. Metals clustering in close proximity exhibit similar behavioral profiles, suggesting comparable modes of neurotoxic action, whereas distantly clustered metals display distinct toxicity patterns. Differences in clustering between species highlight species-specific sensitivity to metal exposure. Heatmaps depict the direction and magnitude of behavioral changes across endpoints, with red indicating increased activity and light blue indicating decreased activity relative to controls. Hierarchical clustering in the heatmaps is consistent with PCA results, grouping metals that induce similar neurobehavioral responses.

**Figure 12:**
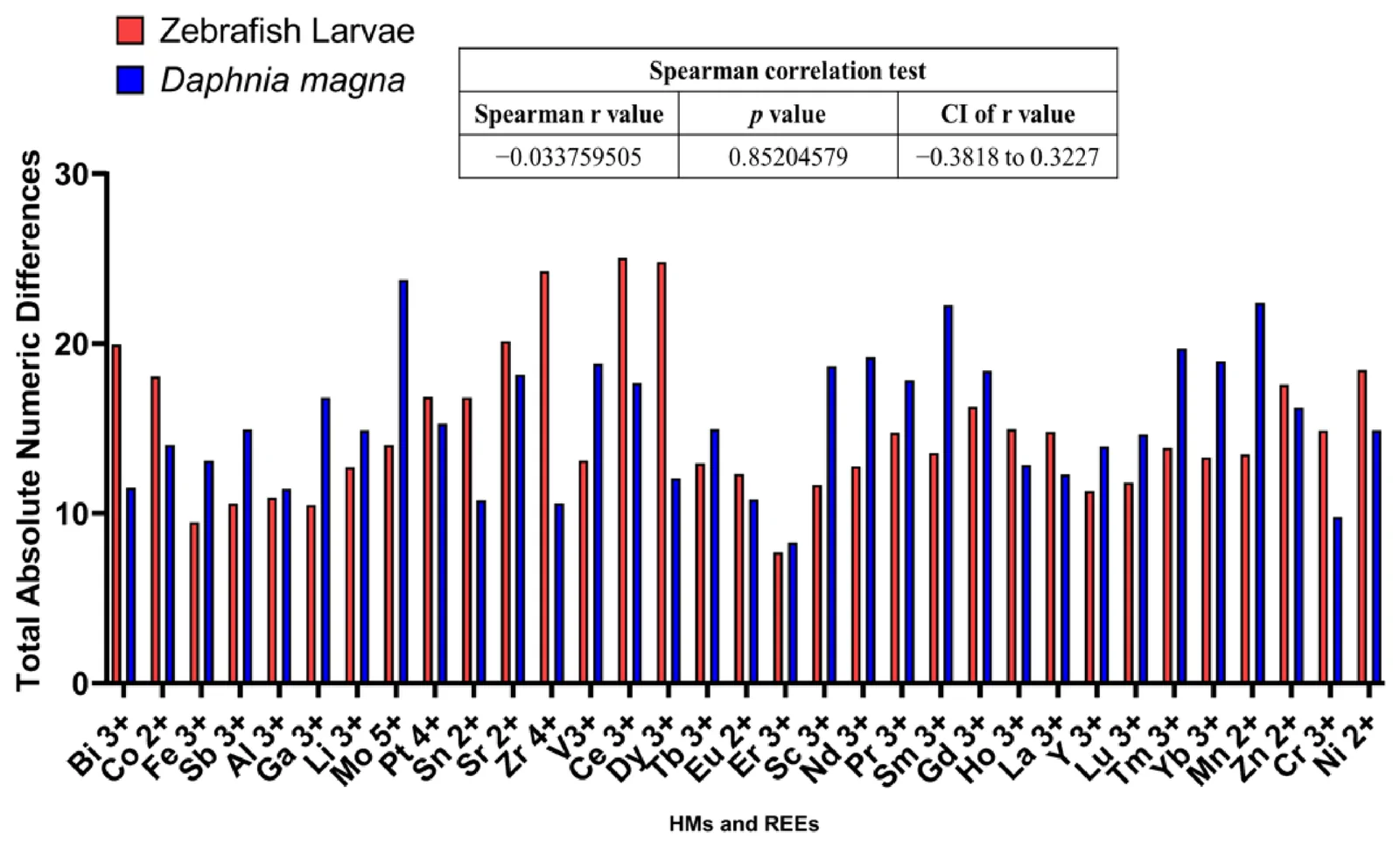
Comparative toxicity assessment between zebrafish larvae and *Daphnia magna.* Spearman correlation analysis illustrates the relationship between toxicity rankings in zebrafish larvae and *Daphnia magna*, highlighting species-dependent differences in metal-induced behavioral responses.

**Table 2:** Details of the HMs and REEs, including their chloride forms and CAS numbers, were used to conduct behavioral assays on both zebrafish larvae and *Daphnia magna*.

HMs and RREs	Chloride Form	CAS Number
Al	Aluminum chloride	7446-70-0
Sb	Antimony trichloride	10025-91-9
Bi	Bismuth trichloride	7787-60-2
Cr	Chromium (III) chloride	10025-73-7
Co	Cobalt (II) chloride	7646-79-9
Ga	Gallium (III) chloride	13450-90-3
Fe	Iron (III) chloride	7705-08-0
Li	Lithium chloride	7447-41-8
Mn	Manganese (II) chloride	7773-01-5
Mo	Molybdenum (V) chloride	10241-05-1
Ni	Nickel (II) chloride	7718-54-9
Pt	Platinum (IV) chloride	13454-96-1
Sr	Strontium chloride	10476-85-4
Sn	Tin (II) chloride	7772-99-8
V	Vanadium (III) chloride	7718-98-1
Zn	Zinc chloride	7646-85-7
Zr	Zirconium (IV) chloride	10026-11-6
Ce	Cerium (III) chloride heptahydrate	18618-55-8
Dy	Dysprosium (III) chloride hexahydrate	15059-52-6
Er	Erbium (III) chloride hexahydrate	10025-75-9
Eu	Europium (III) chloride hexahydrate	13759-92-7
Gd	Gadolinium (III) chloride hexahydrate	13450-84-5
Ho	Holmium (III) chloride hexahydrate	14914-84-2
La	Lanthanum (III) chloride hexahydrate	17272-45-6
Lu	Lutetium (III) chloride hexahydrate	15230-79-2
Nd	Neodymium (III) chloride hexahydrate	13477-89-9
Pr	Praseodymium (III) chloride	10361-79-2
Tb	Terbium (III) chloride hexahydrate	13798-24-8
Tm	Thulium (III) chloride hexahydrate	1331-74-4
Sm	Samarium (III) chloride hexahydrate	13465-55-9
Sc	Scandium (III) chloride	10361-84-9
Yb	Ytterbium (III) chloride	10361-91-8
Y	Yttrium (III) chloride hexahydrate	10025-94-2

## Data Availability

The original data presented in the study are openly available at https://zenodo.org/records/18075567 (accessed on 1 January 2026).

## Supplementary Materials

The following supporting information can be downloaded at https://www.mdpi.com/article/10.3390/toxics14100862/s1, Figure S1: Locomotor activity profiles of zebrafish larvae during light-to-dark and dark-to-light transitions; Figure S2: Burst activity patterns of zebrafish larvae during the transition from light to dark; Figure S3: Rotation patterns of zebrafish larvae during the transition from light to dark; Figure S4: Locomotor activity profiles of *Daphnia magna* during light-to-dark and dark-to-light transitions; Figure S5: Burst activity patterns of *Daphnia magna* during the transition from light to dark; Figure S6: Rotation patterns of *Daphnia magna* during the transition from light to dark. Table S1: Summary of zebrafish larvae and *Daphnia magna* behavioral endpoint; Table S2: The 96 h mortality rates of zebrafish larvae and the 48 h mortality rates of *Daphnia magna* after exposure to HMs and REEs to determine the median lethal concentration (LC50); Table S3: Compares the total distance traveled by HMs and REEs treated zebrafish larvae during light and dark cycles; Table S4: Compares Burst Movement Count by HMs and REEs treated zebrafish larvae during light and dark cycles; Table S5: Compares Rotation Count by HMs and REEs treated zebrafish larvae during light and dark cycles; Table S6: Statistical analysis of locomotor activity of zebrafish larvae after HMs and REEs exposure, four behavioral endpoints were measured as Average speed, Freezing Time Ratio, Swimming Time Ratio, and Rapid movement time ratio; Table S7: Statistical analysis of exploratory activity of zebrafish larvae after HMs and REEs exposure, two behavioral endpoints were measured as Thigmotaxis and Time spent in the center Area; Table S8: Statistical analysis of movement orientation of zebrafish larvae after HMs and REEs exposure, two behavioral endpoints were measured as Average Angular Velocity and Meandering; Table S9: Statistical analysis of movement complexity of zebrafish larvae after HMs and REEs exposure, two behavioral endpoints were measured as Fractal Dimension and Entropy; Table S10: Compares the total distance traveled by HMs and REEs treated *Daphnia magna* during light and dark cycles; Table S11: Compares Burst Movement Count by HMs and REEs treated *Daphnia magna* during light and dark cycles; Table S12: Compares Rotation Count by HMs and REEs treated *Daphnia magna* during light and dark cycles; Table S13: Statistical analysis of locomotor activity of *Daphnia magna* after HMs and REEs exposure, four behavioral endpoints were measured as Average speed, Freezing Time Ratio, Swimming Time Ratio, and Rapid movement time ratio; Table S14: Statistical analysis of exploratory activity of *Daphnia magna* after HMs and REEs exposure, two behavioral endpoints were measured as Thigmotaxis and Time spent in the center Area; Table S15: Statistical analysis of movement orientation of *Daphnia magna* after HMs and REEs exposure, two behavioral endpoints were measured as Average Angular Velocity and Meandering; Table S16: Statistical analysis of movement complexity of *Daphnia magna* after HMs and REEs exposure, two behavioral endpoints were measured as Fractal Dimension and Entropy; Table S17: Ranking index of zebrafish larvae based on heatmap numeric differences; Table S18: Ranking index of *Daphnia magna* based on heatmap numeric differences.
