# Supplementary material for: Comparison of Toxicity of Heavy Metals and Rare Earth Elements in Zebrafish Larvae and Daphnia magna Neonates Using a Phenomics Approach with Multiple Locomotor Activity Endpoints
Source: Toxics. 2026 Sep 28;14(10):862. doi: 10.3390/toxics14100862 (PMC13709712; doi:10.3390/toxics14100862)
Supplement: Supplementary file 1 [file toxics-14-00862-s001.zip › toxics-4512899-supplementary.pdf]

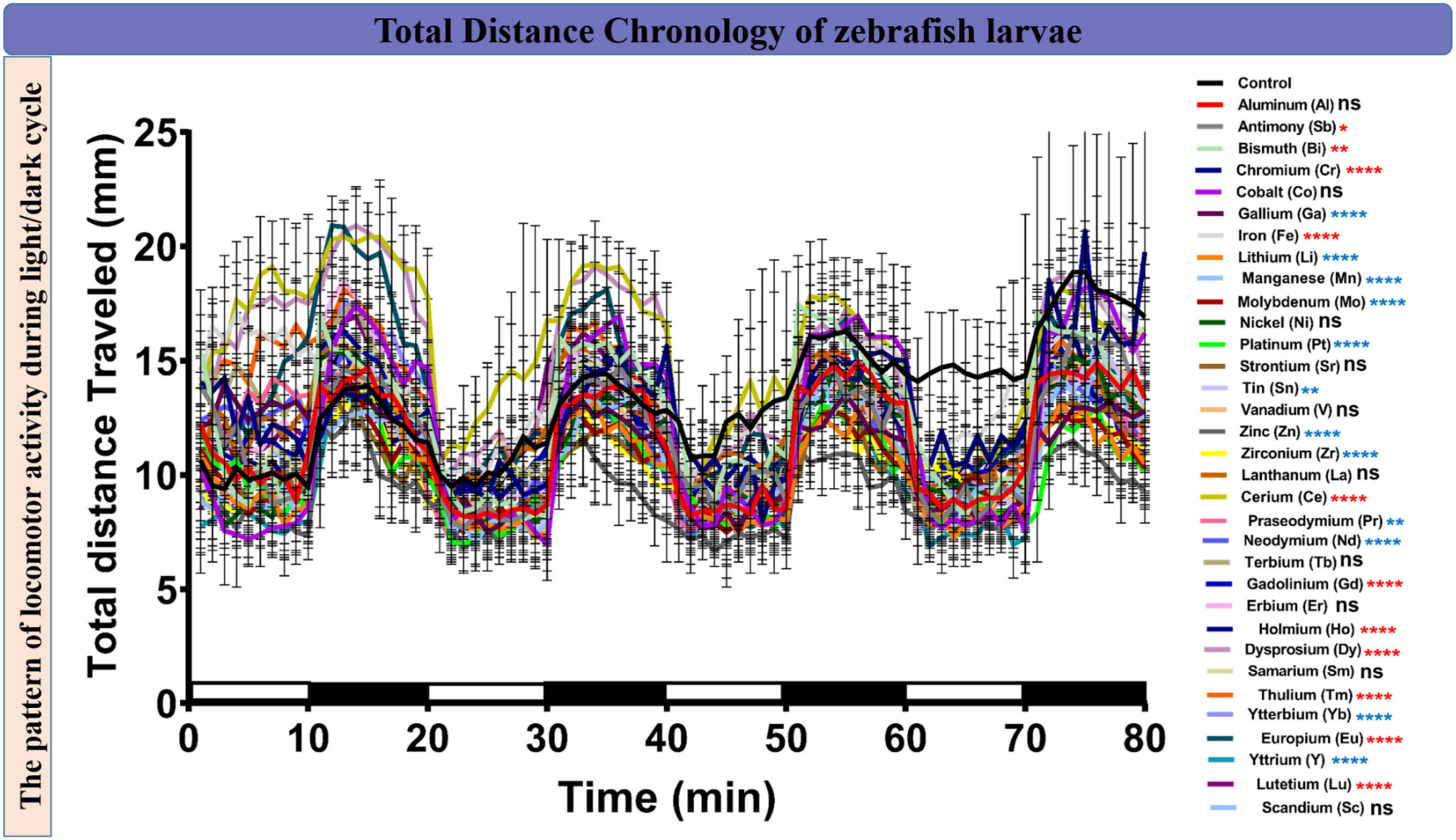

Figure S1. Locomotor activity profiles of zebrafish larvae during light-to-dark and dark-to-light transitions. Data are presented as Mean ± SEM and analyzed using Two-way ANOVA. Dunnett's multiple comparison test was used to compare all treatments with the control and to assess the effects of HMs and REEs. Sample size: n = 48. Statistical significance is indicated as (ns (not significant), *$p < 0.05$, **$p < 0.01$, ***$p < 0.001$, ****$p < 0.0001$).

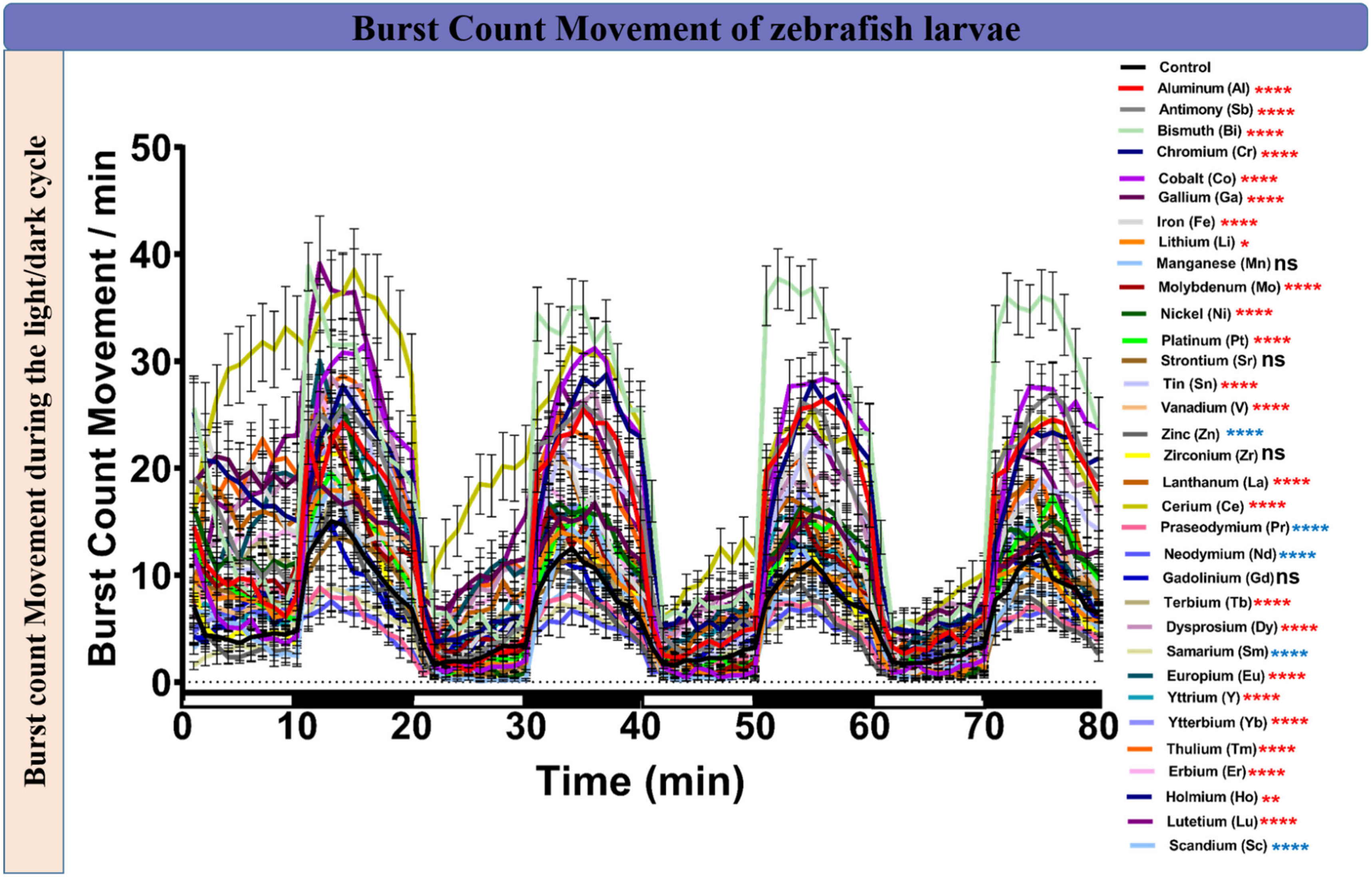

Figure S2. Burst activity patterns of zebrafish larvae during the transition from light to dark. Data are presented as Mean ± SEM and analyzed using Two-way ANOVA. Dunnett's multiple comparison test was used to compare all treatments with the control and to assess

the effects of HMs and REEs. Sample size: n = 48. Statistical significance is indicated as (ns (not significant), *p < 0.05, **p < 0.01, ***p < 0.001, ****p < 0.0001).

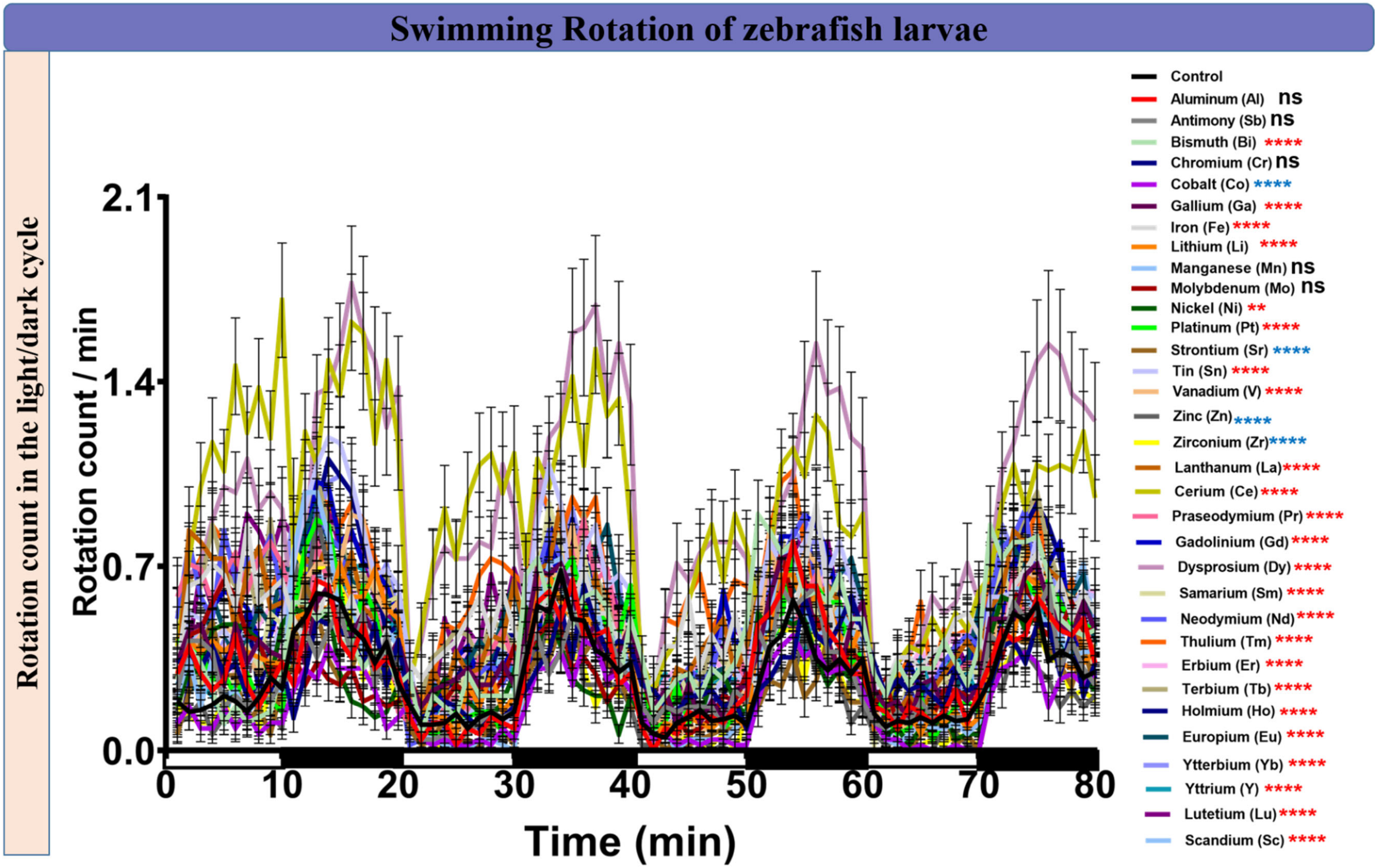

Figure S3. Rotation patterns of zebrafish larvae during the transition from light to dark. Data are presented as Mean ± SEM and analyzed using Two-way ANOVA. Dunnett's multiple comparison test was used to compare all treatments with the control and to assess the effects of HMs and REEs. Sample size: n = 48. Statistical significance is indicated as (ns (not significant), *p < 0.05, **p < 0.01, ***p < 0.001, ****p < 0.0001).

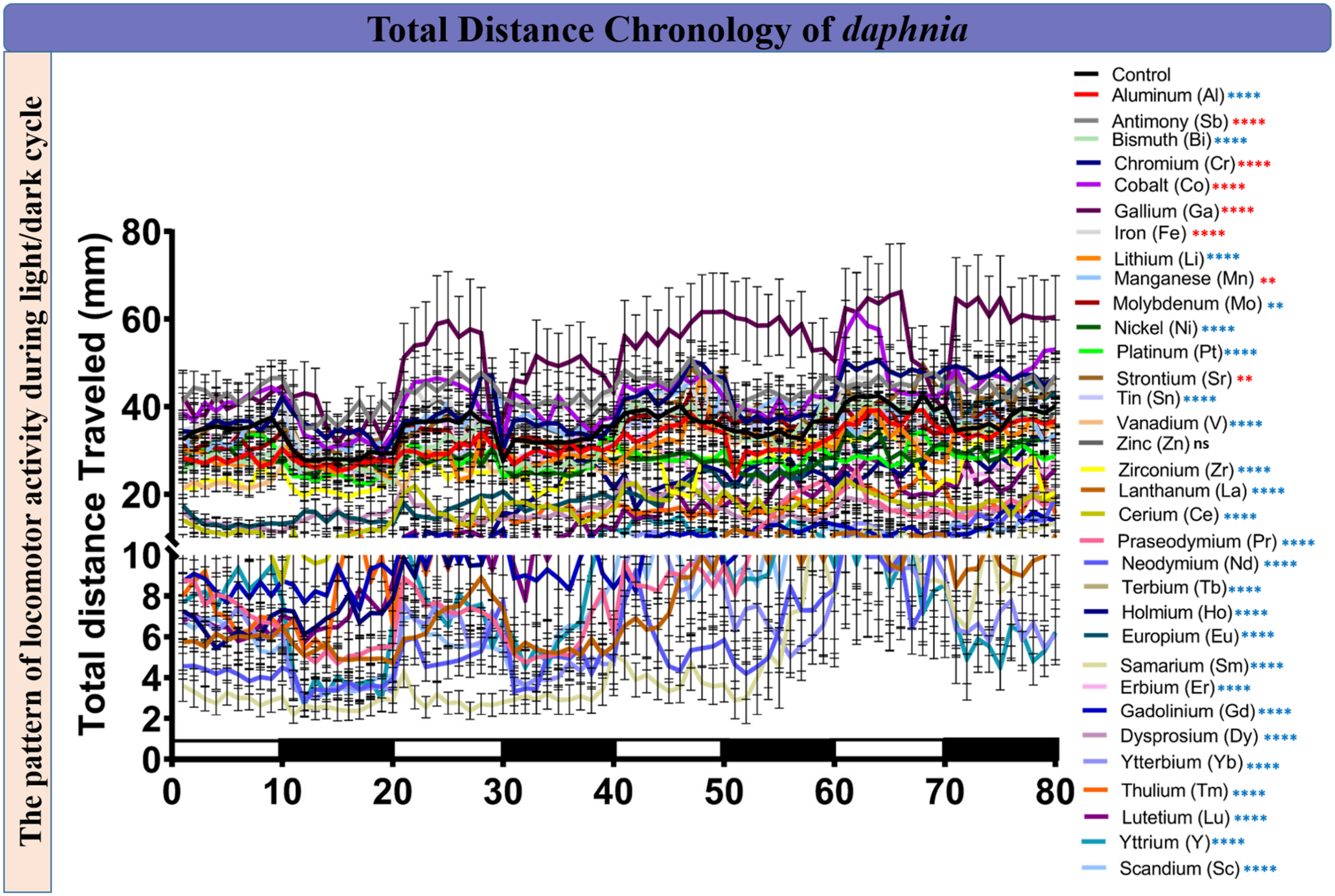

Figure S4. Locomotor activity profiles of *Daphnia magna* during light-to-dark and dark-to-light transitions. Data are presented as Mean ± SEM and analyzed using Two-way ANOVA. Dunnett's multiple comparison test was used to compare all treatments with the control and to assess the effects of HMs and REEs. Sample size: n = 48. Statistical significance is indicated as (ns (not significant), *p < 0.05, **p < 0.01, ***p < 0.001, ****p < 0.0001).

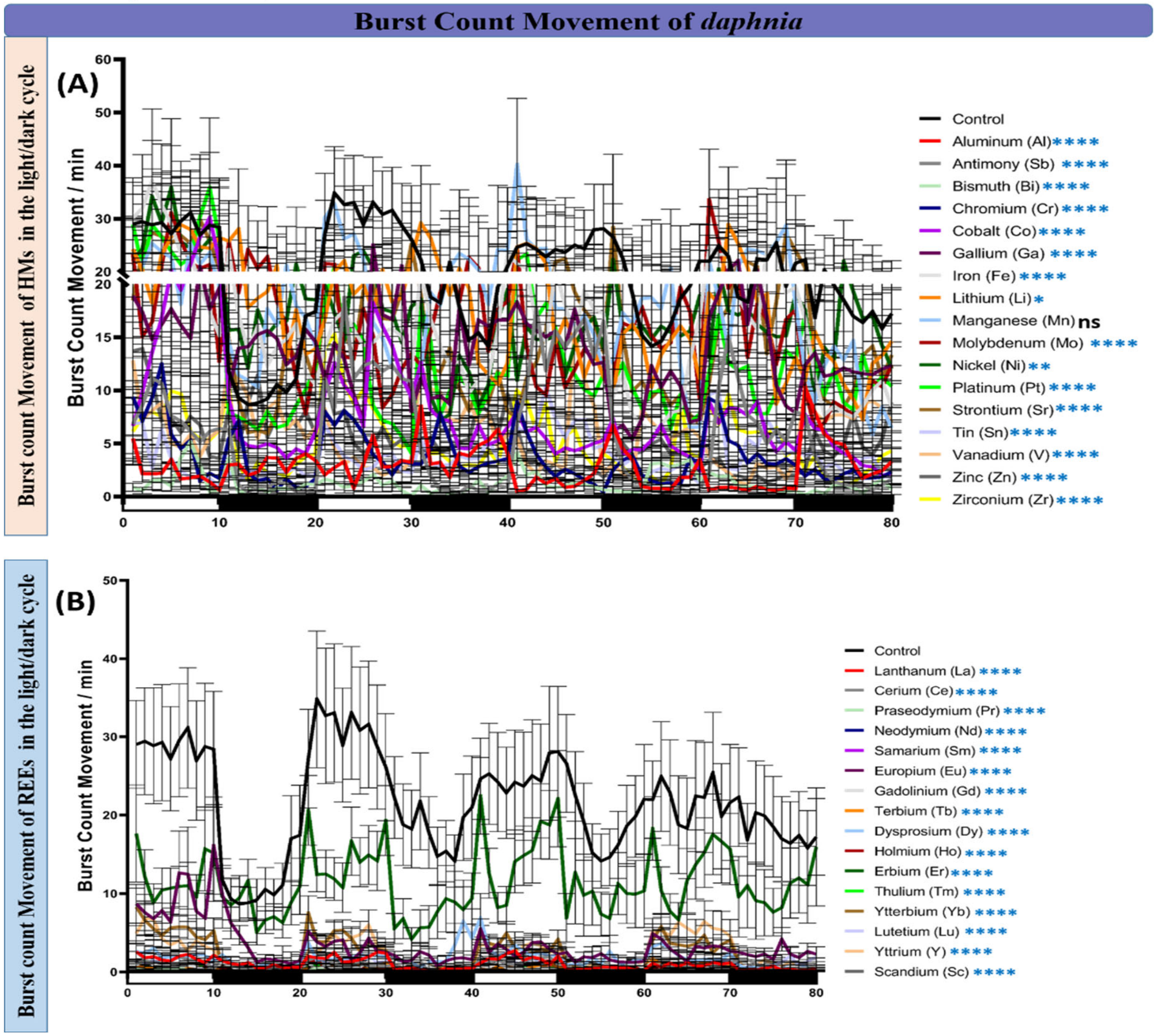

Figure S5. Burst activity patterns of *Daphnia magna* during the transition from light to dark. **(A)** Burst activity of *Daphnia magna* after HMs exposure during the transition from light to dark. **(B)** Burst activity of *Daphnia magna* after REEs exposure during the transition from light to dark Data are presented as Mean ± SEM and analyzed using Two-way ANOVA. Dunnett's multiple comparison test was used to compare all treatments with the control and to assess the effects of HMs and REEs. Sample size: n = 48. Statistical significance is indicated as (ns (not significant), *p < 0.05, **p < 0.01, ***p < 0.001, ****p < 0.0001).

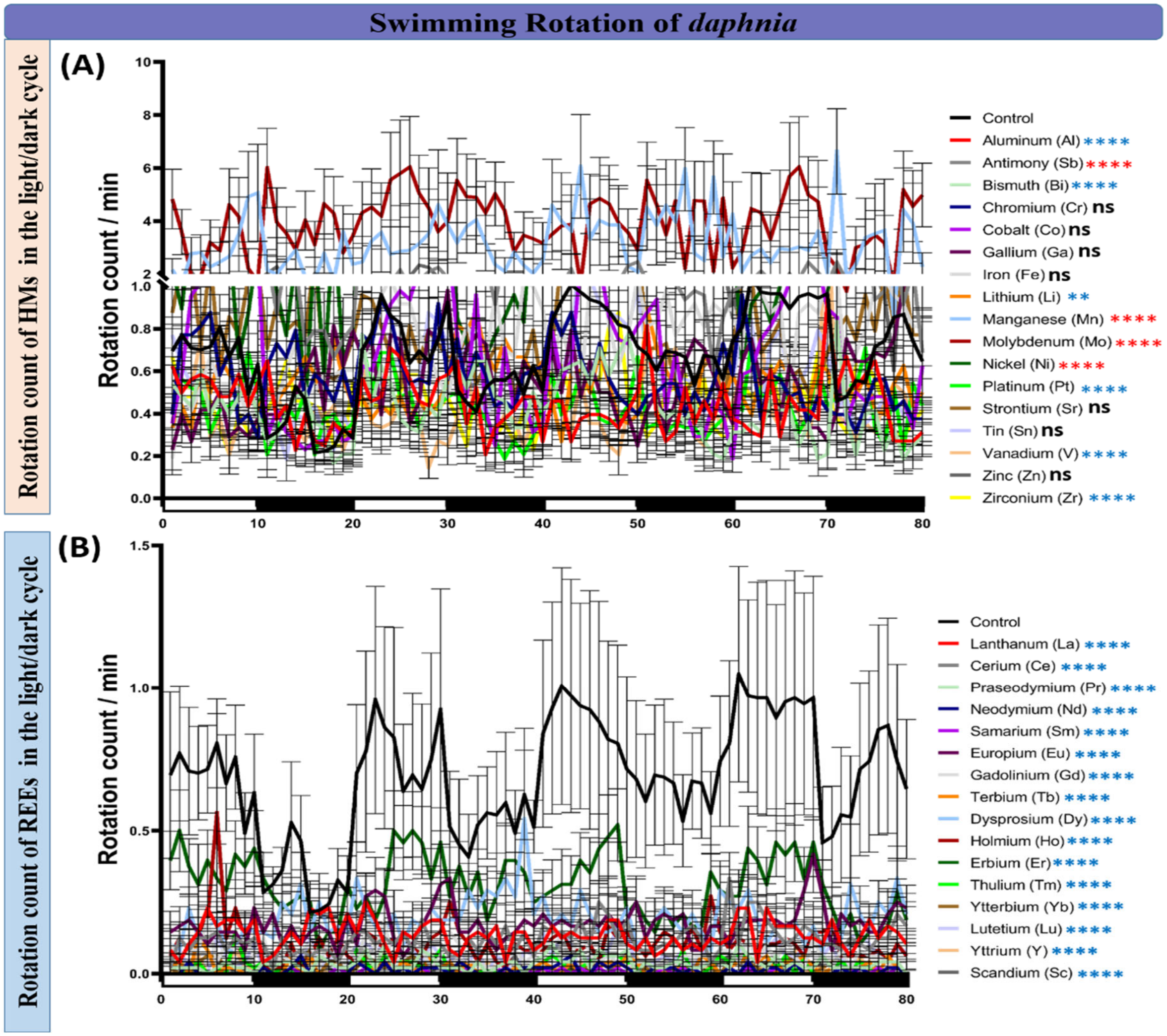

Figure S6. Rotation patterns of *Daphnia magna* during the transition from light to dark. **(A)** Rotation patterns of *Daphnia magna* after HMs exposure during the transition from light to dark. **(B)** Rotation patterns of *Daphnia magna* after REEs exposure during the transition from light to dark. Data are presented as Mean ± SEM and analyzed using Two-way ANOVA. Dunnett's multiple comparison test was used to compare all treatments with the control and to assess the effects of HMs and REEs. Sample size: $n = 48$. Statistical significance is indicated as (ns (not significant), *$p < 0.05$, **$p < 0.01$, ***$p < 0.001$, ****$p < 0.0001$).

Table S1. Summary of zebrafish larvae and *Daphnia magna* behavioral endpoint.

Behavior Endpoint Category	Behavior Endpoints (Units)	Definition
**Locomotor activity**	Total Distance Traveled (mm/s)	Total distance traveled divided by total time duration.
	Burst Count (pixels/s)	The rapid movement during the experiment was measured by counting body movements exceeding 20 pixels per second.
	Rotation (◦/m)	The rotation movement event is defined as the total rotation count that exceeds 0.1 mm in diameter and 60° of back angle per minute.
	Average speed (cm/s)	Total distance traveled divided by total time duration
	Freezing time movement ratio (%)	Total percentage of time when speed is less than 0.5 cm/s.
	Swimming time movement ratio (%)	Total percentage of time when speed is between 1 and 10 cm/s.
	Rapid movement ratio (%)	Total percentage of time when speed is more than 10 cm/s.
**Movement Orientation**	Angular Velocity (◦/s)	The average magnitude at which an animal rotates or revolves about an axis over time.
	Meandering (◦/m)	Movement without a fixed direction.
**Exploratory behavior**	Thigmotaxis (cm)	Average distance to the center of the test tank.
	Time spent in the center area	Total distance traveled in the center of the test tank.
**Movement complexity**	Fractal Dimensions	Complexity of fractal patterns as a ratio of the change in detail to the change proportionally.
	Entropy	The uncertainty and degree of disorder.

Table S2: The 96 h mortality rates of zebrafish larvae and the 48 h mortality rates of *Daphnia magna* after exposure to HMs and REEs to determine the median lethal concentration (LC_{50}).

HMs and REEs	Larvae LC_{50}(mg/L)	Daphnia LC_{50} (mg/L)
**Al**	1.392	0.1
**Sb**	0.1218	0.1039
**Bi**	0.414	0.4731
**Cr**	1.138	0.08914
**Co**	16.77	0.09397
**Ga**	0.1174	0.1139
**Fe**	0.2761	0.08914
**Li**	0.5447	0.1
**Mn**	0.4731	0.1002
**Mo**	0.1285	0.106
**Ni**	0.9878	0.06554
**Pt**	0.1285	0.1
**Sr**	8.077	0.1044
**Sn**	0.4259	0.08914
**V**	0.8783	0.08328
**Zn**	0.8644	Ambiguous*
**Zr**	0.1218	0.09612
**La**	1.29	0.08914
**Ce**	1.17	0.09164
**Pr**	1.215	0.1
**Nd**	0.9425	0.1139
**Sm**	1.083	0.0864
**Eu**	1.17	Ambiguous
**Gd**	1.29	0.09164
**Tb**	1.703	0.106
**Dy**	1.215	0.09814
**Ho**	1.29	0.09814
**Er**	1.29	0.08328
**Tm**	1.334	0.1174

**Yb**	1.695	0.09612
**Lu**	1.17	0.1019
**Y**	1.695	0.09814
**Sc**	0.1109	Ambiguous

*Ambiguous indicates that a reliable LC_{50} value could not be calculated because the mortality/immobilization data did not produce a sufficiently defined concentration-response relationship within the tested concentration range.

Table S3: Compares the total distance traveled by HMs and REEs treated zebrafish larvae during light and dark cycles, analyzed with a mixed-model two-way ANOVA. ns indicates no significant difference, while n/a means not applicable.

Group Comparisons	Total Distance Traveled		Light cycle				Dark cycle			
	Significance	*P* Value	Mean	Std. Deviation	Significance	*P* Value	Mean	Std. Deviation	Significance	*P* Value
**Control**	n/a	n/a	0.7472	3.896	n/a	n/a	0.6191	2.685	n/a	n/a
**Al**	****	<0.0001	0.9036	2.006	****	<0.0001	1.035	1.681	****	<0.0001
**Sb**	****	<0.0001	1.027	2.44	****	<0.0001	0.9813	1.898	****	<0.0001
**Bi**	****	<0.0001	0.4531	0.9428	ns	0.4485	0.4349	0.9056	****	<0.0001
**Cr**	ns	>0.0525	0.4875	1.059	ns	0.1729	0.4448	0.9583	****	<0.0001
**Co**	ns	0.9997	0.6427	2.014	ns	>0.9999	0.6151	2.008	ns	>0.2209
**Ga**	ns	0.0590	0.6573	2.09	ns	>0.9999	0.5901	1.817	ns	>0.9999
**Fe**	ns	0.9856	0.6042	1.689	***	0.0007	0.6016	1.758	****	<0.0001
**Li**	**	0.0014	0.726	2.191	****	<0.0001	0.6505	2.121	****	<0.0001
**Mn**	****	<0.0001	0.376	1.089	****	<0.0001	0.3651	1.012	ns	>0.2223
**Mo**	****	<0.0001	0.3474	1.016	****	<0.0001	0.3443	0.9822	**	<0.0067
**Ni**	****	<0.0001	0.7922	2.372	ns	0.3171	0.7188	2.171	****	<0.0001
**Pt**	****	<0.0001	0.8807	2.811	**	0.0022	0.8109	2.552	****	<0.0001
**Sr**	ns	0.1053	0.688	1.526	****	<0.0001	0.6104	1.479	****	<0.0001
**Sn**	ns	0.2159	0.7786	1.941	****	<0.0001	0.6964	1.869	****	<0.0001
**V**	****	<0.0001	1.057	2.996	****	<0.0001	0.999	3.092	****	<0.0001
**Zn**	****	<0.0001	0.9052	2.49	**	0.0025	0.9047	2.676	****	<0.0001
**Zr**	****	<0.0001	0.4927	1.484	ns	>0.9999	0.4609	1.139	ns	>0.1535
**La**	****	<0.0001	0.09896	0.3828	****	<0.0001	0.1016	0.3717	****	<0.0001
**Ce**	****	<0.0001	0.1635	0.6145	****	<0.0001	0.1458	0.4398	****	<0.0001

**Pr**	****	<0.0001	0.02917	0.1683	****	<0.0001	0.02656	0.1608	****	<0.0001
**Nd**	****	<0.0001	0.02083	0.1465	****	<0.0001	0.02083	0.1429	****	<0.0001
**Sm**	****	<0.0001	0.009375	0.09639	****	<0.0001	0.01042	0.1016	****	<0.0001
**Eu**	****	<0.0001	0.2547	0.6312	****	<0.0001	0.2307	0.5868	****	<0.0001
**Gd**	****	<0.0001	0.1063	0.5452	****	<0.0001	0.0849	0.3603	****	<0.0001
**Tb**	****	<0.0001	0.02292	0.1565	****	<0.0001	0.02188	0.1498	****	<0.0001
**Dy**	****	<0.0001	0.1781	0.5455	****	<0.0001	0.1495	0.4344	****	<0.0001
**Ho**	****	<0.0001	0.1063	0.5461	****	<0.0001	0.07552	0.3667	****	<0.0001
**Er**	****	<0.0001	0.2396	0.5961	****	<0.0001	0.2344	0.6033	****	<0.0001
**Tm**	****	<0.0001	0.03021	0.1772	****	<0.0001	0.02656	0.1608	****	<0.0001
**Yb**	****	<0.0001	0.05521	0.2284	****	<0.0001	0.04167	0.1999	****	<0.0001
**Lu**	****	<0.0001	0.1198	0.4124	****	<0.0001	0.09271	0.3518	****	<0.0001
**Y**	****	<0.0001	0.03542	0.1904	****	<0.0001	0.03438	0.1822	****	<0.0001
**Sc**	****	<0.0001	0.01302	0.1134	****	<0.0001	0.0151	0.122	****	<0.0001

Table S4: Compares Burst Movement Count by HMs and REEs treated zebrafish larvae during light and dark cycles, analyzed with a mixed-model two-way ANOVA. ns indicates no significant difference, while n/a means not applicable.

**Group Comparisons**	**Burst Movement**		**Light cycle**				**Dark cycle**			
	**Significance**	***P* Value**	**Mean**	**Std. Deviation**	**Significance**	***P* Value**	**Mean**	**Std. Deviation**	**Significance**	***P* Value**
**Control**	n/a	n/a	3.007	7.856	n/a	n/a	9.708	11.96	n/a	n/a
**Al**	****	<0.0001	15.66	16.24	****	<0.0001	13.01	15.32	ns	0.0873
**Sb**	****	<0.0001	13.17	15.01	****	<0.0001	13.03	15.64	**	0.0085
**Bi**	****	<0.0001	8.402	12.74	****	<0.0001	31.09	18.61	****	<0.0001
**Cr**	****	<0.0001	9.003	14.03	****	<0.0001	9.423	15.58	****	<0.0001
**Co**	****	<0.0001	2.795	9.001	****	<0.0001	25.18	19.03	****	<0.0001
**Ga**	****	<0.0001	15.8	15.89	****	<0.0001	14.55	16.28	****	<0.0001
**Fe**	****	<0.0001	9.671	11.94	****	<0.0001	10.09	13.07	***	0.0006
**Li**	*	0.0144	9.39	14.76	****	<0.0001	6.818	12.05	****	<0.0001
**Mn**	ns	0.9929	5.451	9.328	****	<0.0001	4.602	9.553	****	<0.0001
**Mo**	****	<0.0001	12.76	16.18	****	<0.0001	9.745	15.41	****	<0.0001
**Ni**	****	<0.0001	7.645	11.88	****	<0.0001	6.082	10.66	****	<0.0001
**Pt**	****	<0.0001	9.481	14.51	****	<0.0001	7.821	14.53	****	<0.0001
**Sr**	ns	0.9992	6.602	11.89	****	<0.0001	5.996	10.84	****	<0.0001
**Sn**	****	<0.0001	11.91	16.8	****	<0.0001	9.929	16.72	****	<0.0001
**V**	****	<0.0001	8.232	12.6	****	<0.0001	8.154	13.77	****	<0.0001
**Zn**	****	<0.0001	8.645	12.78	****	<0.0001	5.31	10.12	****	<0.0001
**Zr**	ns	0.9287	5.303	7.841	****	<0.0001	3.511	6.737	****	<0.0001
**La**	****	<0.0001	16.72	20.36	****	<0.0001	15.97	19.3	****	<0.0001
**Ce**	****	<0.0001	14	16.99	****	<0.0001	13.41	16.45	****	<0.0001

**Pr**	****	<0.0001	3.927	7.817	****	<0.0001	4.145	8.421	****	<0.0001
**Nd**	****	<0.0001	5.338	8.545	****	<0.0001	5.366	8.978	****	<0.0001
**Sm**	****	<0.0001	5.589	9.928	****	<0.0001	5.139	9.452	****	<0.0001
**Eu**	****	<0.0001	13.35	17.71	****	<0.0001	12.9	17	ns	>0.9999
**Gd**	ns	0.9638	7.458	12.13	****	<0.0001	7.516	13.12	****	<0.0001
**Tb**	****	<0.0001	13.95	15.38	****	<0.0001	13.7	15.61	****	<0.0001
**Dy**	****	<0.0001	22.32	21.72	****	<0.0001	21.78	21.87	****	<0.0001
**Ho**	**	0.0045	6.357	10.81	****	<0.0001	6.492	11.48	****	<0.0001
**Er**	****	<0.0001	8.188	12.71	****	<0.0001	8.022	12.41	****	<0.0001
**Tm**	****	<0.0001	12.37	15.32	****	<0.0001	11.98	15.32	****	<0.0001
**Yb**	****	<0.0001	10.97	15.66	****	<0.0001	10.78	15.78	ns	0.0661
**Lu**	****	<0.0001	10.73	15.24	****	<0.0001	10.53	14.61	ns	>0.9999
**Y**	****	<0.0001	8.208	13.03	****	<0.0001	7.682	12.29	****	<0.0001
**Sc**	****	<0.0001	4.507	8.496	****	<0.0001	4.342	8.508	****	<0.0001

Table S5: Compares Rotation Count by HMs and REEs treated zebrafish larvae during light and dark cycles, analyzed with a mixed-model two-way ANOVA. ns indicates no significant difference, while n/a means not applicable.

**Group Comparisons**	**Rotation Count**		**Light cycle**				**Dark cycle**			
	**Significance**	***P* Value**	**Mean**	**Std. Deviation**	**Significance**	***P* Value**	**Mean**	**Std. Deviation**	**Significance**	***P* Value**
**Control**	n/a	n/a	0.1356	0.426	n/a	n/a	0.418	0.7365	n/a	n/a
**Al**	ns	0.1322	0.2682	0.5406	****	<0.0001	0.3276	0.5752	ns	0.0933
**Sb**	ns	0.9986	0.3172	0.5792	****	<0.0001	0.2781	0.5589	****	<0.0001
**Bi**	****	<0.0001	0.2536	0.5457	****	<0.0001	0.5776	0.722	****	<0.0001
**Cr**	ns	0.3123	0.3302	0.7097	****	<0.0001	0.3167	0.6258	****	<0.0001
**Co**	****	<0.0001	0.05781	0.2486	****	<0.0001	0.2911	0.5326	****	<0.0001
**Ga**	****	<0.0001	0.451	0.7486	****	<0.0001	0.4438	0.719	ns	0.5117
**Fe**	****	<0.0001	0.4068	0.7541	****	<0.0001	0.4073	0.7437	ns	>0.9999
**Li**	****	<0.0001	0.4177	0.8508	****	<0.0001	0.3443	0.6661	**	0.0024
**Mn**	ns	0.1934	0.3073	0.6759	****	<0.0001	0.262	0.6348	****	<0.0001
**Mo**	**	0.0029	0.3797	0.7044	****	<0.0001	0.2906	0.6201	****	<0.0001
**Ni**	ns	0.9994	0.3021	0.612	****	<0.0001	0.2385	0.5415	****	<0.0001
**Pt**	****	<0.0001	0.3557	0.7154	****	<0.0001	0.2755	0.6198	****	<0.0001
**Sr**	****	<0.0001	0.2573	0.5877	****	<0.0001	0.2495	0.5809	****	<0.0001
**Sn**	****	<0.0001	0.5219	0.8903	****	<0.0001	0.4589	0.8334	ns	>0.9999
**V**	****	<0.0001	0.3766	0.6964	****	<0.0001	0.3641	0.6727	ns	0.2523
**Zn**	****	<0.0001	0.2833	0.6293	****	<0.0001	0.2333	0.5791	****	<0.0001
**Zr**	****	<0.0001	0.09896	0.3286	ns	>0.9999	0.3422	0.6506	*	0.021
**La**	****	<0.0001	0.5542	0.8597	****	<0.0001	0.5839	0.8506	****	<0.0001
**Ce**	****	<0.0001	0.8026	1.158	****	<0.0001	0.7854	1.075	****	<0.0001

**Pr**	****	<0.0001	0.3813	0.7498	****	<0.0001	0.3854	0.7897	ns	0.1618
**Nd**	****	<0.0001	0.5036	0.7993	****	<0.0001	0.4901	0.8368	ns	0.1595
**Sm**	****	<0.0001	0.4422	0.7123	****	<0.0001	0.4182	0.6973	ns	>0.9999
**Eu**	****	<0.0001	0.501	0.862	****	<0.0001	0.4927	0.8443	ns	0.6211
**Gd**	****	<0.0001	0.4833	0.8429	****	<0.0001	0.488	0.8553	ns	0.387
**Tb**	****	<0.0001	0.6333	0.8051	****	<0.0001	0.6599	0.8054	****	<0.0001
**Dy**	****	<0.0001	1.069	1.174	****	<0.0001	1.075	1.252	****	<0.0001
**Ho**	****	<0.0001	0.3979	0.7255	****	<0.0001	0.4552	0.7948	ns	>0.9999
**Er**	****	<0.0001	0.4026	0.7653	****	<0.0001	0.3943	0.815	**	0.0097
**Tm**	****	<0.0001	0.4641	0.7108	****	<0.0001	0.501	0.7418	****	<0.0001
**Yb**	****	<0.0001	0.4516	0.7706	****	<0.0001	0.4635	0.8146	ns	>0.9999
**Lu**	****	<0.0001	0.4229	0.7314	****	<0.0001	0.4016	0.6653	ns	>0.9999
**Y**	****	<0.0001	0.401	0.7684	****	<0.0001	0.3932	0.7654	ns	>0.9999
**Sc**	****	<0.0001	0.4182	0.7114	****	<0.0001	0.3771	0.6628	ns	>0.9999

Table S6: Statistical analysis of locomotor activity of zebrafish larvae after HMs and REEs exposure, four behavioral endpoints were measured as Average speed, Freezing Time Ratio, Swimming Time Ratio, and Rapid movement time ratio. These were analyzed with a mixed-model two-way ANOVA. ns indicates no significant difference, while 'n/a' means not applicable.

**Group Comparisons**	**Average speed**				**Freezing Time Ratio**				**Swimming Time Ratio**				**Rapid movement time ratio**			
	**Mean**	**Std. Deviation**	**Significance**	***P* Value**	**Mean**	**Std. Deviation**	**Significance**	***P* Value**	**Mean**	**Std. Deviation**	**Significance**	***P* Value**	**Mean**	**Std. Deviation**	**Significance**	***P* Value**
**Control**	3.174	1.032	n/a	n/a	81.92	7.17	n/a	n/a	16.90	12.75	n/a	n/a	0.9361	1.001	n/a	n/a
**Al**	2.468	0.996	ns	0.9008	86.46	7.081	ns	>0.9999	6.535	6.498	ns	>0.9999	0.7889	0.8629	ns	>0.9999
**Sb**	2.27	0.8606	ns	0.1673	88.18	5.919	ns	0.2540	16.90	12.75	ns	0.2598	0.8111	0.6106	ns	>0.9999
**Bi**	3.09	0.7614	ns	>0.9999	84.26	5.197	ns	>0.9999	6.535	6.498	ns	0.3815	1.35	0.6243	ns	0.6617

**Cr**	2.058	0.9287	*	0.0239	90.72	5.634	**	0.0022	16.90	12.75	**	0.0012	0.9611	1.064	ns	>0.9999
**Co**	2.47	0.9459	ns	>0.9999	87.78	6.427	ns	0.3876	6.535	6.498	ns	0.4306	0.8611	0.8154	ns	>0.9999
**Ga**	2.559	0.921	ns	>0.9999	86.99	5.807	ns	>0.9999	16.90	12.75	ns	>0.9999	0.75	0.489	ns	>0.9999
**Fe**	2.125	0.8366	*	0.0374	89.14	5.698	ns	0.0557	6.535	6.498	ns	0.1575	0.3111	0.2948	ns	0.3595
**Li**	2.469	0.6248	ns	>0.9999	86.97	5.072	ns	>0.9999	16.90	12.75	ns	>0.9999	0.3556	0.1917	ns	>0.9999
**Mn**	1.977	0.7998	**	0.0051	90.36	5.603	**	0.0049	6.535	6.498	**	0.0059	0.6444	0.6455	ns	>0.9999
**Mo**	2.719	0.8994	ns	>0.9999	85.31	5.469	ns	>0.9999	16.90	12.75	ns	>0.9999	0.9611	0.8204	ns	>0.9999
**Ni**	1.887	0.5292	***	0.0007	90.57	3.713	**	0.0026	6.535	6.498	**	0.0034	0.7167	0.478	ns	>0.9999
**Pt**	3.259	0.8637	ns	>0.9999	82.63	5.196	ns	>0.9999	16.90	12.75	ns	>0.9999	1.194	0.8721	ns	>0.9999
**Sr**	3.03	1.191	ns	>0.9999	83.12	7.688	ns	>0.9999	6.535	6.498	ns	>0.9999	0.8111	1.158	ns	>0.9999
**Sn**	2.936	0.5656	ns	>0.9999	83.25	4.436	ns	>0.9999	16.90	12.75	ns	>0.9999	1.05	0.6706	ns	>0.9999
**V**	2.843	0.694	ns	>0.9999	84.49	4.485	ns	>0.9999	6.535	6.498	ns	>0.9999	1.272	1.037	ns	>0.9999
**Zn**	1.768	0.836	***	0.0003	91.36	5.559	***	0.0005	16.90	12.75	**	0.0011	0.4222	0.2798	ns	>0.9999
**Zr**	3.553	0.6558	ns	>0.9999	80.14	4.693	ns	>0.9999	6.535	6.498	ns	>0.9999	0.9944	0.6646	ns	>0.9999
**La**	2.235	1.545	ns	0.1598	89.92	9.698	**	0.0038	16.90	12.75	***	0.0003	1.978	1.514	ns	0.2392
**Ce**	2.865	1.136	ns	>0.9999	85.44	7.773	ns	>0.9999	6.535	6.498	ns	>0.9999	2.111	1.111	**	0.0058
**Pr**	2.01	1.475	*	0.0140	91.56	7.95	***	0.0004	16.90	12.75	****	<0.0001	1.728	1.479	ns	>0.9999
**Nd**	2.301	0.7838	ns	0.2303	90.31	4.955	**	0.0044	6.535	6.498	***	0.0003	1.967	1.203	*	0.0305
**Sm**	2.014	1.386	*	0.0115	91.37	7.23	***	0.0005	16.90	12.75	****	<0.0001	1.517	1.411	ns	>0.9999
**Eu**	2.174	0.6656	*	0.0488	91.03	3.855	***	0.0009	6.535	6.498	****	<0.0001	1.978	0.7353	**	0.0016

**Gd**	2.015	0.8403	**	0.0081	92.59	3.84	****	<0.0001	16.90	12.75	****	<0.0001	1.95	1.28	ns	0.0502
**Tb**	2.614	1.033	ns	>0.9999	89.14	5.53	ns	0.0549	6.535	6.498	**	0.0025	2.261	1.381	**	0.0050
**Dy**	2.474	1.165	ns	>0.9999	87.17	7.807	ns	0.8613	16.90	12.75	ns	0.4649	1.417	1.28	ns	>0.9999
**Ho**	1.922	1.354	**	0.0017	87.03	9.959	ns	0.5184	6.535	6.498	****	<0.0001	1.333	1.015	ns	>0.9999
**Er**	2.106	0.5221	*	0.0116	91.92	3.5	****	<0.0001	16.90	12.75	****	<0.0001	1.744	0.6836	*	0.0106
**Tm**	1.907	0.6847	**	0.0013	92.03	3.904	****	<0.0001	6.535	6.498	****	<0.0001	1.428	0.7537	ns	0.4382
**Yb**	1.746	0.9433	***	0.0001	91.88	7.503	****	<0.0001	16.90	12.75	****	<0.0001	1.017	0.5659	ns	>0.9999
**Lu**	2.04	1.199	*	0.0110	90.42	7.643	**	0.0025	6.535	6.498	***	0.0009	1.256	1.26	ns	>0.9999
**Y**	2.152	0.986	ns	0.0564	89.99	7.035	**	0.0067	16.90	12.75	***	0.0009	1.544	0.9847	ns	0.3995
**Sc**	2.217	1.308	ns	0.1155	90.98	6.414	**	0.0012	6.535	6.498	****	<0.0001	1.883	1.448	ns	0.3912

Table S7: Statistical analysis of exploratory activity of zebrafish larvae after HMs and REEs exposure, two behavioral endpoints were measured as Thigmotaxis and Time spent in the center Area. These were analyzed with a mixed-model two-way ANOVA. ns indicates no significant difference, while n/a means not applicable.

**Group Comparisons**	**Thigmotaxis**				**Time spent in center Area**			
	**Mean**	**Std. Deviation**	**Significance**	***P* Value**	**Mean**	**Std. Deviation**	**Significance**	***P* Value**
**Control**	12.57	4.006	n/a	n/a	27.27	11.19	n/a	n/a
**Al**	12.88	3.66	ns	>0.9999	29.02	20.85	ns	>0.9999
**Sb**	12.99	4.174	ns	>0.9999	26.75	13.97	ns	>0.9999
**Bi**	12.26	2.941	ns	>0.9999	29.82	13.34	ns	>0.9999
**Cr**	15.17	3.279	ns	0.2773	18.33	19.72	ns	0.2637

**Co**	13.97	2.641	ns	>0.9999	21.57	14.44	ns	>0.9999
**Ga**	13.24	3.282	ns	>0.9999	30.24	14.75	ns	>0.9999
**Fe**	12.02	2.404	ns	>0.9999	29.8	17.05	ns	>0.9999
**Li**	11.82	3.378	ns	>0.9999	30.8	19.68	ns	>0.9999
**Mn**	13.16	3.945	ns	>0.9999	17	13.89	ns	0.4284
**Mo**	11.54	2.684	ns	>0.9999	32.34	13.54	ns	>0.9999
**Ni**	15.6	1.329	*	0.0154	12.2	10.63	**	0.0050
**Pt**	11.97	2.219	ns	>0.9999	28.46	11.73	ns	>0.9999
**Sr**	11.55	2.4	ns	>0.9999	38.09	12.74	ns	0.3076
**Sn**	11.7	2.212	ns	>0.9999	34.08	9.839	ns	>0.9999
**V**	12.74	1.946	ns	>0.9999	23.62	9.559	ns	>0.9999
**Zn**	14.48	2.556	ns	>0.9999	15.78	14.28	ns	0.2175
**Zr**	12.78	1.894	ns	>0.9999	32.12	11.55	ns	>0.9999
**La**	14.64	2.307	ns	>0.9999	19.38	23.65	ns	0.1777
**Ce**	11.87	2.303	ns	>0.9999	31.82	9.899	ns	>0.9999
**Pr**	12.59	2.937	ns	>0.9999	19.52	14.82	ns	>0.9999
**Nd**	13.28	1.801	ns	>0.9999	21.83	12.85	ns	>0.9999
**Sm**	13.25	2.597	ns	>0.9999	17.32	13.13	ns	0.4446
**Eu**	11.82	1.932	ns	>0.9999	28.43	12.22	ns	>0.9999
**Gd**	13.1	2.37	ns	>0.9999	17	12.33	ns	0.3043
**Tb**	11.97	1.647	ns	>0.9999	27.27	10.94	ns	>0.9999
**Dy**	12.52	2.601	ns	>0.9999	21.83	13.43	ns	>0.9999
**Ho**	13.67	2.062	ns	>0.9999	23.35	21.33	ns	>0.9999

**Er**	12.83	1.711	ns	>0.9999	23.37	9.535	ns	>0.9999
**Tm**	12.66	2.242	ns	>0.9999	29.18	10.54	ns	>0.9999
**Yb**	13.14	3.058	ns	>0.9999	25.55	18.01	ns	>0.9999
**Lu**	14.03	1.774	ns	>0.9999	16.74	11.66	ns	0.2392
**Y**	14.74	5.171	ns	>0.9999	20.62	12.15	ns	>0.9999
**Sc**	13.59	2.403	ns	>0.9999	27.21	22.08	ns	>0.9999

Table S8: Statistical analysis of movement orientation of zebrafish larvae after HMs and REEs exposure, two behavioral endpoints were measured as Average Angular Velocity and Meandering. These were analyzed with a mixed-model two-way ANOVA. ns indicates no significant difference, while n/a means not applicable.

**Group Comparisons**	**Average Angular Velocity**				**Meandering**			
	**Mean**	**Std. Deviation**	**Significance**	***P* Value**	**Mean**	**Std. Deviation**	**Significance**	***P* Value**
**Control**	6.994	1.509	n/a	n/a	49.96	19.32	n/a	n/a
**Al**	7.753	1.801	ns	>0.9999	70.13	30.18	ns	>0.9999
**Sb**	8.67	1.939	ns	0.9094	69.65	19.21	ns	0.9923
**Bi**	7.621	1.271	ns	>0.9999	50.35	18.09	ns	>0.9999
**Cr**	10.08	4.298	ns	0.5103	109.5	77.87	*	0.0334
**Co**	8.858	3.185	ns	>0.9999	84.51	60.72	ns	>0.9999
**Ga**	7.885	2.333	ns	>0.9999	73.84	47.94	ns	>0.9999
**Fe**	9.256	3.286	ns	>0.9999	89.69	40.13	*	0.0404
**Li**	7.788	2.05	ns	>0.9999	78.53	30.43	ns	0.2721
**Mn**	9.856	2.748	ns	0.0892	93.95	60.54	ns	0.1376
**Mo**	7.629	1.456	ns	>0.9999	57.99	29.91	ns	>0.9999

**Ni**	9.197	1.825	ns	0.1209	70.55	37.4	ns	>0.9999
**Pt**	6.854	1.61	ns	>0.9999	45.63	31.23	ns	>0.9999
**Sr**	7.025	1.314	ns	>0.9999	62.2	27.47	ns	>0.9999
**Sn**	7.179	0.8759	ns	>0.9999	54.17	13.24	ns	>0.9999
**V**	7.29	1.462	ns	>0.9999	52.6	29.82	ns	>0.9999
**Zn**	10.51	3.381	*	0.0385	95.09	45.2	*	0.0180
**Zr**	6.181	1.366	ns	>0.9999	43.22	29.08	ns	>0.9999
**La**	14.4	7.761	**	0.0030	91.37	71.15	ns	>0.9999
**Ce**	10.52	6.67	ns	>0.9999	65.35	54.36	ns	>0.9999
**Pr**	13.61	7.333	**	0.0094	75.27	76.03	ns	>0.9999
**Nd**	12.03	3.761	***	0.0008	82.84	66.9	ns	>0.9999
**Sm**	14.63	6.889	***	0.0001	96.6	64.65	ns	0.3917
**Eu**	12.4	4.498	***	0.0009	74.28	46.4	ns	>0.9999
**Gd**	14.91	2.772	****	<0.0001	118.7	61.4	***	0.0006
**Tb**	12.13	4.208	***	0.0006	88.87	80.63	ns	>0.9999
**Dy**	9.714	4.759	ns	>0.9999	83.83	82.59	ns	>0.9999
**Ho**	15.25	7.143	***	0.0002	118.8	83.53	*	0.0407
**Er**	11.53	3.556	**	0.0020	62.59	28.5	ns	>0.9999
**Tm**	16	4.119	****	<0.0001	108.4	35.62	****	<0.0001
**Yb**	14.48	5.307	****	<0.0001	115	63.82	**	0.0016
**Lu**	12.71	5.248	**	0.0057	121	86.87	ns	0.0994
**Y**	13.27	5	****	<0.0001	86.79	31.41	*	0.0323
**Sc**	15.01	8.396	***	0.0004	94.53	67.85	ns	0.7990

Table S9: Statistical analysis of movement complexity of zebrafish larvae after HMs and REEs exposure, two behavioral endpoints were measured as Fractal Dimension and Entropy. These were analyzed with a mixed-model two-way ANOVA. ns indicates no significant difference, while n/a means not applicable.

**Group Comparisons**	**Fractal Dimension**				**Entropy**			
	**Mean**	**Std. Deviation**	**Significance**	***P* Value**	**Mean**	**Std. Deviation**	**Significance**	***P* Value**
**Control**	0.4324	0.1758	n/a	n/a	0.9761	0.08382	n/a	n/a
**Al**	0.4256	0.2163	ns	>0.9999	0.9506	0.07163	ns	>0.9999
**Sb**	0.428	0.2293	ns	>0.9999	0.9464	0.1078	ns	>0.9999
**Bi**	0.5132	0.2158	ns	>0.9999	0.9727	0.06805	ns	>0.9999
**Cr**	0.332	0.2111	ns	>0.9999	0.8985	0.1108	ns	0.4271
**Co**	0.4077	0.2281	ns	>0.9999	0.9371	0.07172	ns	>0.9999
**Ga**	0.4446	0.2259	ns	>0.9999	0.9148	0.1889	ns	>0.9999
**Fe**	0.4667	0.2493	ns	>0.9999	0.9189	0.1144	ns	>0.9999
**Li**	0.5576	0.2187	ns	>0.9999	0.926	0.06594	ns	0.2532
**Mn**	0.3908	0.1888	ns	>0.9999	0.9222	0.1254	ns	>0.9999
**Mo**	0.4936	0.1578	ns	>0.9999	0.9693	0.1283	ns	>0.9999
**Ni**	0.2137	0.1222	**	0.0086	0.8149	0.1975	**	0.0044
**Pt**	0.5566	0.1616	ns	>0.9999	1.003	0.03913	ns	>0.9999
**Sr**	0.5901	0.161	ns	0.9217	0.9609	0.06708	ns	>0.9999
**Sn**	0.5795	0.06127	ns	0.7344	1.023	0.01687	ns	0.6022
**V**	0.5317	0.1564	ns	>0.9999	0.9779	0.05821	ns	>0.9999
**Zn**	0.3491	0.2275	ns	>0.9999	0.88	0.172	ns	0.5987
**Zr**	0.6287	0.2082	ns	0.5679	0.9716	0.04511	ns	>0.9999

**La**	0.2293	0.1872	*	0.0158	0.8489	0.2398	ns	>0.9999
**Ce**	0.3128	0.1245	ns	>0.9999	0.9806	0.0741	ns	>0.9999
**Pr**	0.1982	0.1449	**	0.0029	0.8392	0.2477	ns	0.2878
**Nd**	0.213	0.09954	**	0.0082	0.9005	0.1969	ns	>0.9999
**Sm**	0.2045	0.133	**	0.0048	0.8563	0.2054	ns	>0.9999
**Eu**	0.258	0.08125	ns	0.1965	0.9629	0.09232	ns	>0.9999
**Gd**	0.1655	0.09453	***	0.0002	0.9165	0.1249	ns	>0.9999
**Tb**	0.243	0.08853	ns	0.0622	0.9473	0.08761	ns	>0.9999
**Dy**	0.2892	0.1321	ns	0.9168	0.9207	0.2105	ns	>0.9999
**Ho**	0.2229	0.2036	**	0.0078	0.8426	0.1911	*	0.0317
**Er**	0.2373	0.09165	*	0.0490	0.9247	0.1278	ns	>0.9999
**Tm**	0.227	0.1493	*	0.0219	0.9356	0.1593	ns	>0.9999
**Yb**	0.2111	0.2212	**	0.0031	0.8569	0.1403	*	0.0163
**Lu**	0.2322	0.1536	*	0.0264	0.8841	0.2397	ns	>0.9999
**Y**	0.2952	0.2268	ns	0.6099	0.851	0.237	ns	>0.9999
**Sc**	0.2476	0.1586	ns	0.0652	0.9367	0.09067	ns	>0.9999

Table S10: Compares the total distance traveled by HMs and REEs treated *Daphnia magna* during light and dark cycles, analyzed with a mixed-model two-way ANOVA. ns indicates no significant difference, while n/a means not applicable.

Group Comparisons	Total Distance Traveled		Light cycle				Dark cycle			
	Significance	*P* Value	Mean	Std. Deviation	Significance	*P* Value	Mean	Std. Deviation	Significance	*P* Value
**Control**	n/a	n/a	29.19	23.38	n/a	n/a	28.63	35.48	n/a	n/a
**Al**	****	<0.0001	35.01	23.12	****	<0.0001	25.66	23.42	****	<0.0001
**Sb**	****	<0.0001	38.16	27.71	****	<0.0001	28.63	35.48	****	<0.0001
**Bi**	****	<0.0001	31.39	21.63	****	<0.0001	25.66	23.42	****	<0.0001
**Cr**	****	<0.0001	39.79	30.67	****	<0.0001	28.63	35.48	****	<0.0001
**Co**	****	<0.0001	41.66	40.97	****	<0.0001	25.66	23.42	****	<0.0001
**Ga**	****	<0.0001	51.05	51.58	****	<0.0001	28.63	35.48	****	<0.0001
**Fe**	****	<0.0001	32.58	31.98	****	<0.0001	25.66	23.42	****	<0.0001
**Li**	****	<0.0001	37.03	31.08	****	<0.0001	28.63	35.48	****	<0.0001
**Mn**	**	0.0013	32.38	19.48	****	<0.0001	25.66	23.42	****	<0.0001
**Mo**	**	0.0071	37.64	20.27	****	<0.0001	28.63	35.48	****	<0.0001
**Ni**	****	<0.0001	27.35	19.7	ns	>0.9999	25.66	23.42	ns	0.1010
**Pt**	****	<0.0001	28.1	18.65	ns	>0.9999	28.63	35.48	ns	0.5685
**Sr**	**	0.0050	30.07	26.15	ns	>0.9999	25.66	23.42	***	0.0001
**Sn**	****	<0.0001	38.11	30.15	****	<0.0001	28.63	35.48	****	<0.0001
**V**	****	<0.0001	26.98	28.22	****	<0.0001	25.66	23.42	ns	0.0763
**Zn**	ns	0.9996	32.3	28.02	ns	>0.9999	28.63	35.48	****	<0.0001
**Zr**	****	<0.0001	27.19	18	ns	>0.9999	25.66	23.42	ns	>0.9999
**La**	****	<0.0001	8.014	23.56	****	<0.0001	28.63	35.48	****	<0.0001
**Ce**	****	<0.0001	20.45	37.1	****	<0.0001	25.66	23.42	****	<0.0001

**Pr**	****	<0.0001	10.97	34.68	****	<0.0001	28.63	35.48	****	<0.0001
**Nd**	****	<0.0001	5.064	8.124	****	<0.0001	25.66	23.42	****	<0.0001
**Sm**	****	<0.0001	3.846	6.132	****	<0.0001	28.63	35.48	****	<0.0001
**Eu**	****	<0.0001	18.03	26.19	****	<0.0001	25.66	23.42	****	<0.0001
**Gd**	****	<0.0001	7.333	17.15	****	<0.0001	28.63	35.48	****	<0.0001
**Tb**	****	<0.0001	7.546	15.68	****	<0.0001	25.66	23.42	****	<0.0001
**Dy**	****	<0.0001	12.82	18.22	****	<0.0001	28.63	35.48	****	<0.0001
**Ho**	****	<0.0001	16.09	41.75	****	<0.0001	25.66	23.42	****	<0.0001
**Er**	****	<0.0001	30.45	46.68	****	<0.0001	28.63	35.48	****	<0.0001
**Tm**	****	<0.0001	13.87	28.81	****	<0.0001	25.66	23.42	****	<0.0001
**Yb**	****	<0.0001	5.294	11.61	****	<0.0001	28.63	35.48	****	<0.0001
**Lu**	****	<0.0001	9.527	25.79	****	<0.0001	25.66	23.42	****	<0.0001
**Y**	****	<0.0001	8.658	21.54	****	<0.0001	28.63	35.48	****	<0.0001
**Sc**	****	<0.0001	8.149	22.23	****	<0.0001	25.66	23.42	****	<0.0001

Table S11: Compares Burst Movement Count by HMs and REEs treated *Daphnia magna* during light and dark cycles, analyzed with a mixed-model two-way ANOVA. ns indicates no significant difference, while n/a means not applicable.

Group Comparisons	Burst Movement		Light cycle				Dark cycle			
	Significance	*P* Value	Mean	Std. Deviation	Significance	*P* Value	Mean	Std. Deviation	Significance	*P* Value
**Control**	n/a	n/a	25.9	69.34	n/a	n/a	16.70	2.472	n/a	n/a
**Al**	****	<0.0001	8.879	38.98	****	<0.0001	47.61	14.15	****	<0.0001
**Sb**	****	<0.0001	9.542	46.6	****	<0.0001	16.70	2.472	****	<0.0001
**Bi**	****	<0.0001	13.31	41.83	**	0.0018	47.61	14.15	****	<0.0001
**Cr**	****	<0.0001	10.44	34.55	****	<0.0001	16.70	2.472	****	<0.0001
**Co**	****	<0.0001	19.57	52.24	ns	>0.9999	47.61	14.15	****	<0.0001
**Ga**	****	<0.0001	14.3	44.03	ns	0.0853	16.70	2.472	****	<0.0001
**Fe**	****	<0.0001	20.1	56.7	****	<0.0001	47.61	14.15	****	<0.0001
**Li**	*	0.0113	15.83	48.53	*	0.0119	16.70	2.472	ns	0.0561
**Mn**	ns	0.0832	18.35	51.4	ns	>0.9999	47.61	14.15	ns	>0.9999
**Mo**	****	<0.0001	15.16	51.51	**	0.0013	16.70	2.472	*	0.0263
**Ni**	**	0.0020	16.83	49.3	****	<0.0001	47.61	14.15	****	<0.0001
**Pt**	****	<0.0001	14.34	45.19	****	<0.0001	16.70	2.472	****	<0.0001
**Sr**	****	<0.0001	10.06	41.03	****	<0.0001	47.61	14.15	****	<0.0001
**Sn**	****	<0.0001	10.26	42.55	****	<0.0001	16.70	2.472	****	<0.0001
**V**	****	<0.0001	24.16	73.53	****	<0.0001	47.61	14.15	****	<0.0001
**Zn**	****	<0.0001	23.89	76.98	****	<0.0001	16.70	2.472	****	<0.0001
**Zr**	****	<0.0001	7.117	21.49	****	<0.0001	47.61	14.15	****	<0.0001
**La**	****	<0.0001	1.01	3.593	****	<0.0001	16.70	2.472	****	<0.0001
**Ce**	****	<0.0001	1.449	6.847	****	<0.0001	47.61	14.15	****	<0.0001

**Pr**	****	<0.0001	0.04531	0.3617	****	<0.0001	16.70	2.472	****	<0.0001
**Nd**	****	<0.0001	0.07969	0.5614	****	<0.0001	47.61	14.15	****	<0.0001
**Sm**	****	<0.0001	0.1365	1.717	****	<0.0001	16.70	2.472	****	<0.0001
**Eu**	****	<0.0001	8.795	22.77	ns	>0.9999	47.61	14.15	ns	>0.9999
**Gd**	****	<0.0001	0.6698	4.571	****	<0.0001	16.70	2.472	****	<0.0001
**Tb**	****	<0.0001	0.3615	1.492	****	<0.0001	47.61	14.15	****	<0.0001
**Dy**	****	<0.0001	1.514	7.455	****	<0.0001	16.70	2.472	****	<0.0001
**Ho**	****	<0.0001	0.4	2.706	****	<0.0001	47.61	14.15	****	<0.0001
**Er**	****	<0.0001	8.389	24.63	*	0.0358	16.70	2.472	****	<0.0001
**Tm**	****	<0.0001	0.3724	1.546	****	<0.0001	47.61	14.15	****	<0.0001
**Yb**	****	<0.0001	2.545	7.603	****	<0.0001	16.70	2.472	****	<0.0001
**Lu**	****	<0.0001	0.9302	3.912	****	<0.0001	47.61	14.15	****	<0.0001
**Y**	****	<0.0001	2.402	7.929	****	<0.0001	16.70	2.472	****	<0.0001
**Sc**	****	<0.0001	0.1875	1.935	****	<0.0001	47.61	14.15	****	<0.0001

Table S12: Compares Rotation Count by HMs and REEs treated *Daphnia magna* during light and dark cycles, analyzed with a mixed-model two-way ANOVA. ns indicates no significant difference, while n/a means not applicable.

**Group Comparisons**	**Rotation Count**		**Light cycle**				**Dark cycle**			
	**Significance**	***P* Value**	**Mean**	**Std. Deviation**	**Significance**	***P* Value**	**Mean**	**Std. Deviation**	**Significance**	***P* Value**
**Control**	n/a	n/a	0.7472	3.896	n/a	n/a	0.6191	2.685	n/a	n/a
**Al**	****	<0.0001	0.9036	2.006	****	<0.0001	1.035	1.681	****	<0.0001
**Sb**	****	<0.0001	1.027	2.44	****	<0.0001	0.9813	1.898	****	<0.0001
**Bi**	****	<0.0001	0.4531	0.9428	ns	0.4485	0.4349	0.9056	****	<0.0001
**Cr**	ns	0.0525	0.4875	1.059	ns	0.1729	0.4448	0.9583	****	<0.0001
**Co**	ns	0.9997	0.6427	2.014	ns	>0.9999	0.6151	2.008	ns	0.2209
**Ga**	ns	0.0590	0.6573	2.09	ns	>0.9999	0.5901	1.817	ns	>0.9999
**Fe**	ns	0.9856	0.6042	1.689	***	0.0007	0.6016	1.758	****	<0.0001
**Li**	**	0.0014	0.726	2.191	****	<0.0001	0.6505	2.121	****	<0.0001
**Mn**	****	<0.0001	0.376	1.089	****	<0.0001	0.3651	1.012	ns	0.2223
**Mo**	****	<0.0001	0.3474	1.016	****	<0.0001	0.3443	0.9822	**	0.0067
**Ni**	****	<0.0001	0.7922	2.372	ns	0.3171	0.7188	2.171	****	<0.0001
**Pt**	****	<0.0001	0.8807	2.811	**	0.0022	0.8109	2.552	****	<0.0001
**Sr**	ns	0.1053	0.688	1.526	****	<0.0001	0.6104	1.479	****	<0.0001
**Sn**	ns	0.2159	0.7786	1.941	****	<0.0001	0.6964	1.869	****	<0.0001
**V**	****	<0.0001	1.057	2.996	****	<0.0001	0.999	3.092	****	<0.0001
**Zn**	****	<0.0001	0.9052	2.49	**	0.0025	0.9047	2.676	****	<0.0001
**Zr**	****	<0.0001	0.4927	1.484	ns	>0.9999	0.4609	1.139	ns	0.1535
**La**	****	<0.0001	0.09896	0.3828	****	<0.0001	0.1016	0.3717	****	<0.0001
**Ce**	****	<0.0001	0.1635	0.6145	****	<0.0001	0.1458	0.4398	****	<0.0001

**Pr**	****	<0.0001	0.02917	0.1683	****	<0.0001	0.02656	0.1608	****	<0.0001
**Nd**	****	<0.0001	0.02083	0.1465	****	<0.0001	0.02083	0.1429	****	<0.0001
**Sm**	****	<0.0001	0.009375	0.09639	****	<0.0001	0.01042	0.1016	****	<0.0001
**Eu**	****	<0.0001	0.2547	0.6312	****	<0.0001	0.2307	0.5868	****	<0.0001
**Gd**	****	<0.0001	0.1063	0.5452	****	<0.0001	0.0849	0.3603	****	<0.0001
**Tb**	****	<0.0001	0.02292	0.1565	****	<0.0001	0.02188	0.1498	****	<0.0001
**Dy**	****	<0.0001	0.1781	0.5455	****	<0.0001	0.1495	0.4344	****	<0.0001
**Ho**	****	<0.0001	0.1063	0.5461	****	<0.0001	0.07552	0.3667	****	<0.0001
**Er**	****	<0.0001	0.2396	0.5961	****	<0.0001	0.2344	0.6033	****	<0.0001
**Tm**	****	<0.0001	0.03021	0.1772	****	<0.0001	0.02656	0.1608	****	<0.0001
**Yb**	****	<0.0001	0.05521	0.2284	****	<0.0001	0.04167	0.1999	****	<0.0001
**Lu**	****	<0.0001	0.1198	0.4124	****	<0.0001	0.09271	0.3518	****	<0.0001
**Y**	****	<0.0001	0.03542	0.1904	****	<0.0001	0.03438	0.1822	****	<0.0001
**Sc**	****	<0.0001	0.01302	0.1134	****	<0.0001	0.0151	0.122	****	<0.0001

Table S13: Statistical analysis of locomotor activity of *Daphnia magna* after HMs and REEs exposure, four behavioral endpoints were measured as Average speed, Freezing Time Ratio, Swimming Time Ratio, and Rapid movement time ratio. These were analyzed with a mixed-model two-way ANOVA. ns indicates no significant difference, while 'n/a' means not applicable.

**Group Comparisons**	**Average speed**				**Freezing Time Ratio**				**Swimming Time Ratio**				**Rapid movement time ratio**			
	**Mean**	**Std. Deviation**	**Significance**	***P* Value**	**Mean**	**Std. Deviation**	**Significance**	***P* Value**	**Mean**	**Std. Deviation**	**Significance**	***P* Value**	**Mean**	**Std. Deviation**	**Significance**	***P* Value**
**Control**	3.239	0.7917	n/a	n/a	83.31	5.805	n/a	n/a	15.86	4.192	n/a	n/a	1.492	0.9203	n/a	n/a
**Al**	3.113	0.9696	ns	>0.9999	86.12	7.417	ns	>0.9999	12.11	6.204	ns	0.4777	1.778	1.911	ns	>0.9999
**Sb**	2.777	0.5954	ns	>0.9999	88.25	4.859	ns	0.2611	9.756	4.865	**	0.0062	1.994	1.492	ns	>0.9999
**Bi**	3.175	1.54	ns	>0.9999	86.09	10.22	ns	0.5518	11.4	8.596	*	0.0349	2.5	2.249	ns	>0.9999

**Cr**	2.935	1.319	ns	>0.9999	85.74	9.348	ns	>0.9999	13.05	8.226	ns	0.9431	1.189	1.322	ns	>0.9999
**Co**	3.185	0.869	ns	>0.9999	84.67	7.154	ns	>0.9999	14.15	6.284	ns	>0.9999	1.178	1.207	ns	>0.9999
**Ga**	3.546	1.144	ns	>0.9999	81.71	9.474	ns	>0.9999	16.55	8.358	ns	>0.9999	1.767	1.264	ns	>0.9999
**Fe**	3.248	1.338	ns	>0.9999	83.75	11.18	ns	>0.9999	14.58	9.866	ns	>0.9999	1.667	1.764	ns	>0.9999
**Li**	3.012	0.7963	ns	>0.9999	85.01	6.831	ns	>0.9999	14.3	6.414	ns	>0.9999	0.7056	0.5713	ns	0.3129
**Mn**	2.583	0.9807	ns	0.4188	88.29	6.718	ns	0.3460	10.94	5.698	ns	0.2223	0.7722	1.491	*	0.0362
**Mo**	4.227	2.64	ns	>0.9999	75.28	18.86	ns	>0.9999	21.66	14.49	ns	>0.9999	3.061	4.615	ns	>0.9999
**Ni**	3.358	1.445	ns	>0.9999	82.66	11.21	ns	>0.9999	15.36	9.671	ns	>0.9999	1.972	1.845	ns	>0.9999
**Pt**	3.537	1.946	ns	>0.9999	82.28	16.48	ns	>0.9999	16.24	15.31	ns	>0.9999	1.483	1.398	ns	>0.9999
**Sr**	2.33	0.6802	*	0.0159	91.18	5.132	***	0.0005	7.9	4.92	****	<0.0001	0.9278	0.6497	ns	>0.9999
**Sn**	2.947	0.7401	ns	>0.9999	86.91	6.249	ns	>0.9999	11.73	5.626	ns	0.5267	1.372	1.289	ns	>0.9999
**V**	4.012	2.34	ns	>0.9999	83.67	10.1	ns	>0.9999	12.64	6.264	ns	>0.9999	3.678	4.842	ns	>0.9999
**Zn**	2.963	1.031	ns	>0.9999	87.9	5.222	ns	0.4686	11.04	4.762	ns	0.1692	1.039	0.8161	ns	>0.9999
**Zr**	2.759	1.097	ns	>0.9999	86.51	9.236	ns	0.8076	12.95	8.68	ns	0.6554	0.55	0.5924	*	0.0238
**La**	3.112	0.8103	ns	>0.9999	85.09	7.17	ns	>0.9999	13.47	6.79	ns	>0.9999	1.439	1.049	ns	>0.9999
**Ce**	3.873	1.18	ns	>0.9999	82.22	6.836	ns	>0.9999	14.68	5.344	ns	>0.9999	3.089	2.547	ns	>0.9999
**Pr**	3.108	0.8998	ns	>0.9999	84.77	8.07	ns	>0.9999	14.26	7.35	ns	>0.9999	0.9833	1.183	ns	0.9858
**Nd**	3.567	1.252	ns	>0.9999	82.49	9.014	ns	>0.9999	16.26	8.185	ns	>0.9999	1.256	1.005	ns	>0.9999
**Sm**	3.809	0.9429	ns	>0.9999	80.31	7.297	ns	>0.9999	17.31	6.638	ns	>0.9999	2.378	1.673	ns	>0.9999
**Eu**	2.647	0.8443	ns	0.8588	86.13	19.6	*	0.0246	8.056	2.977	***	0.0003	1.428	1.317	ns	>0.9999

**Gd**	3.026	1.32	ns	>0.9999	86.98	7.154	ns	>0.9999	11.03	5.569	ns	0.3938	1.972	2.196	ns	>0.9999
**Tb**	3.427	1.361	ns	>0.9999	82.78	10.19	ns	>0.9999	15.61	8.795	ns	>0.9999	1.594	1.959	ns	>0.9999
**Dy**	3.032	0.8701	ns	>0.9999	86.76	6.413	ns	>0.9999	12.23	6.014	ns	0.7096	1.011	0.838	ns	>0.9999
**Ho**	3.306	1.572	ns	>0.9999	82.39	13.5	ns	>0.9999	15.69	12.29	ns	0.8213	1.889	1.511	ns	>0.9999
**Er**	3.02	1.038	ns	>0.9999	84.98	7.261	ns	>0.9999	13.39	6.778	ns	>0.9999	1.628	1.137	ns	>0.9999
**Tm**	2.828	1.097	ns	>0.9999	89.6	4.768	*	0.0477	7.994	3.892	***	0.0002	2.394	2.025	ns	>0.9999
**Yb**	3.13	1.392	ns	>0.9999	89.53	5.935	*	0.0335	9.311	5.656	**	0.0033	1.4	1.727	ns	>0.9999
**Lu**	3.116	0.6878	ns	>0.9999	87.41	4.07	ns	>0.9999	10.21	3.147	ns	0.0755	2.378	1.491	ns	>0.9999
**Y**	3.018	1.165	ns	>0.9999	88.56	6.306	ns	0.2738	8.939	4.374	**	0.0042	2.511	2.824	ns	>0.9999
**Sc**	2.905	1.326	ns	>0.9999	86.34	8.179	ns	>0.9999	11.92	6.632	ns	0.4557	1.744	2.264	ns	>0.9999

Table S14: Statistical analysis of exploratory activity of *Daphnia magna* after HMs and REEs exposure, two behavioral endpoints were measured as Thigmotaxis and Time spent in the center Area. These were analyzed with a mixed-model two-way ANOVA. ns indicates no significant difference, while n/a means not applicable.

**Group Comparisons**	**Thigmotaxis**				**Time spent in center Area**			
	**Mean**	**Std. Deviation**	**Significance**	***P* Value**	**Mean**	**Std. Deviation**	**Significance**	***P* Value**
**Control**	11.67	3.617	n/a	n/a	39.58	18.81	n/a	n/a
**Al**	11.69	1.67	ns	>0.9999	40.63	14.12	ns	>0.9999
**Sb**	11.75	1.989	ns	>0.9999	40.24	13.9	ns	>0.9999
**Bi**	12.02	2.845	ns	>0.9999	32	11.66	ns	>0.9999
**Cr**	11.54	2.599	ns	>0.9999	35.16	17.17	ns	>0.9999

**Co**	11.46	2.497	ns	>0.9999	42.76	21.31	ns	>0.9999
**Ga**	12.5	2.375	ns	>0.9999	35.4	15.65	ns	>0.9999
**Fe**	10.44	2.847	ns	0.9474	38.47	14.12	ns	>0.9999
**Li**	12.65	2.686	ns	>0.9999	40.76	14.59	ns	>0.9999
**Mn**	12.46	2.476	ns	>0.9999	45.76	21.5	ns	>0.9999
**Mo**	12.4	1.842	ns	>0.9999	35.43	16.36	ns	>0.9999
**Ni**	11.54	2.191	ns	>0.9999	36.95	18.81	ns	>0.9999
**Pt**	11.01	1.869	ns	>0.9999	48.59	18.94	ns	>0.9999
**Sr**	12.21	2.215	ns	>0.9999	40.96	17.57	ns	>0.9999
**Sn**	11.45	2.779	ns	>0.9999	38.54	26.36	ns	>0.9999
**V**	11.79	2.325	ns	>0.9999	44.75	19.94	ns	>0.9999
**Zn**	11.6	3.387	ns	>0.9999	36.04	13.85	ns	>0.9999
**Zr**	10.41	2.138	ns	0.2341	41.11	22.62	ns	>0.9999
**La**	10.8	1.684	ns	0.8220	33.34	9.98	ns	>0.9999
**Ce**	12.56	1.735	ns	>0.9999	29.01	6.466	ns	>0.9999
**Pr**	10.01	3.845	ns	>0.9999	45.09	32.44	ns	>0.9999
**Nd**	12.24	2.911	ns	>0.9999	34.26	12.78	ns	>0.9999
**Sm**	12.54	1.411	ns	>0.9999	29.48	7.776	ns	>0.9999
**Eu**	12.73	1.93	ns	>0.9999	35.55	10.77	ns	>0.9999
**Gd**	14.17	10.68	ns	>0.9999	33.42	17.08	ns	>0.9999
**Tb**	11.34	2.981	ns	>0.9999	39.42	23.15	ns	>0.9999
**Dy**	11.95	2.55	ns	>0.9999	33.81	12.36	ns	>0.9999
**Ho**	11.61	1.362	ns	>0.9999	38.01	16.12	ns	>0.9999

**Er**	12.29	2.139	ns	>0.9999	27.48	10.48	ns	>0.9999
**Tm**	12.71	1.544	ns	>0.9999	37.63	16.51	ns	>0.9999
**Yb**	10.12	3.707	ns	>0.9999	50.29	26.96	ns	>0.9999
**Lu**	11.65	2.811	ns	>0.9999	36.77	17.59	ns	>0.9999
**Y**	11.93	2.038	ns	>0.9999	38.33	22.17	ns	>0.9999
**Sc**	9.678	3.013	ns	0.2334	45.11	25.42	ns	>0.9999

Table S15: Statistical analysis of movement orientation of *Daphnia magna* after HMs and REEs exposure, two behavioral endpoints were measured as Average Angular Velocity and Meandering. These were analyzed with a mixed-model two-way ANOVA. ns indicates no significant difference, while n/a means not applicable.

**Group Comparisons**	**Average Angular Velocity**				**Meandering**			
	**Mean**	**Std. Deviation**	**Significance**	***P* Value**	**Mean**	**Std. Deviation**	**Significance**	***P* Value**
**Control**	5.626	0.9571	n/a	n/a	42.71	12.99	n/a	n/a
**Al**	6.652	1.045	ns	0.2339	49.75	18.13	ns	>0.9999
**Sb**	6.261	1.29	ns	>0.9999	42.05	15.25	ns	>0.9999
**Bi**	6.651	1.823	ns	>0.9999	49.98	29.37	ns	>0.9999
**Cr**	6.253	1.798	ns	>0.9999	48.61	34.58	ns	>0.9999
**Co**	5.238	0.9831	ns	>0.9999	43	15.84	ns	>0.9999
**Ga**	5.442	0.9803	ns	>0.9999	40.11	14.06	ns	>0.9999
**Fe**	5.84	1.547	ns	>0.9999	44.85	24.39	ns	>0.9999
**Li**	5.719	1	ns	>0.9999	50.27	19.56	ns	>0.9999
**Mn**	7.056	2.169	ns	0.6922	77.46	54.32	ns	0.2925
**Mo**	5.654	2.058	ns	>0.9999	45.91	27.59	ns	>0.9999

**Ni**	6.513	3.139	ns	>0.9999	71.02	87.71	ns	>0.9999
**Pt**	5.156	1.693	ns	>0.9999	35.83	23.82	ns	>0.9999
**Sr**	7.026	1.85	ns	0.0723	64.48	45.5	ns	0.7229
**Sn**	6.358	1.622	ns	>0.9999	55.32	29.66	ns	>0.9999
**V**	5.491	0.8678	ns	>0.9999	36.67	19.34	ns	>0.9999
**Zn**	5.304	0.9355	ns	>0.9999	38.97	17.16	ns	>0.9999
**Zr**	5.866	1.762	ns	>0.9999	58.43	34.5	ns	>0.9999
**La**	5.99	1.063	ns	>0.9999	46.53	14.76	ns	>0.9999
**Ce**	5.653	1.045	ns	>0.9999	36.67	15.1	ns	>0.9999
**Pr**	5.307	0.9658	ns	>0.9999	43.48	22.28	ns	>0.9999
**Nd**	5.159	1.104	ns	>0.9999	38.43	16.37	ns	>0.9999
**Sm**	5.404	1.258	ns	>0.9999	33.67	11.51	ns	>0.9999
**Eu**	6.552	1.22	ns	0.8727	55.91	19.24	ns	>0.9999
**Gd**	6.86	2.286	ns	>0.9999	57.37	42.35	ns	>0.9999
**Tb**	5.387	1.654	ns	>0.9999	43.08	28.25	ns	>0.9999
**Dy**	5.331	0.7934	ns	>0.9999	38.48	14.18	ns	>0.9999
**Ho**	5.732	1.552	ns	>0.9999	40.45	21.05	ns	>0.9999
**Er**	6.312	2.62	ns	>0.9999	43.22	35.32	ns	>0.9999
**Tm**	6.422	1.306	ns	>0.9999	32.64	14.86	ns	>0.9999
**Yb**	6.268	1.417	ns	>0.9999	46.7	21.02	ns	>0.9999
**Lu**	6.773	2.209	ns	>0.9999	37.46	14.59	ns	>0.9999
**Y**	6.247	0.9389	ns	>0.9999	43.42	17.01	ns	>0.9999
**Sc**	6.581	1.944	ns	>0.9999	59.53	35.54	ns	>0.9999

Table S16: Statistical analysis of movement complexity of *Daphnia magna* after HMs and REEs exposure, two behavioral endpoints were measured as Fractal Dimension and Entropy. These were analyzed with a mixed-model two-way ANOVA. ns indicates no significant difference, while n/a means not applicable.

**Group Comparisons**	**Fractal Dimension**				**Entropy**			
	**Mean**	**Std. Deviation**	**Significance**	***P* Value**	**Mean**	**Std. Deviation**	**Significance**	***P* Value**
**Control**	0.5066	0.149	n/a	n/a	1.01	0.0437	n/a	n/a
**Al**	0.4044	0.1315	ns	>0.9999	1.003	0.06093	ns	>0.9999
**Sb**	0.394	0.1211	ns	>0.9999	0.9891	0.05977	ns	>0.9999
**Bi**	0.4528	0.1771	ns	>0.9999	0.9367	0.11	ns	>0.1204
**Cr**	0.4452	0.1742	ns	>0.9999	0.9604	0.1351	ns	>0.9999
**Co**	0.5699	0.1588	ns	>0.9999	1.006	0.04173	ns	>0.9999
**Ga**	0.4672	0.1439	ns	>0.9999	1.013	0.04377	ns	>0.9999
**Fe**	0.5562	0.1636	ns	>0.9999	0.9931	0.0488	ns	>0.9999
**Li**	0.5654	0.1162	ns	>0.9999	1.007	0.04236	ns	>0.9999
**Mn**	0.4137	0.161	ns	>0.9999	0.9804	0.06987	ns	>0.9999
**Mo**	0.4929	0.218	ns	>0.9999	0.9737	0.1259	ns	>0.9999
**Ni**	0.4766	0.1587	ns	>0.9999	1.021	0.04469	ns	>0.9999
**Pt**	0.4687	0.148	ns	>0.9999	1	0.05399	ns	>0.9999
**Sr**	0.4079	0.1534	ns	>0.9999	0.9781	0.08615	ns	>0.9999
**Sn**	0.4905	0.1159	ns	>0.9999	1.017	0.03484	ns	>0.9999
**V**	0.4184	0.1458	ns	>0.9999	0.973	0.1072	ns	>0.9999
**Zn**	0.512	0.1899	ns	>0.9999	0.9832	0.05881	ns	>0.9999
**Zr**	0.5627	0.1672	ns	>0.9999	1.009	0.04201	ns	>0.9999

**La**	0.5136	0.1228	ns	>0.9999	0.9928	0.05782	ns	>0.9999
**Ce**	0.5344	0.128	ns	>0.9999	1.033	0.01798	ns	>0.9999
**Pr**	0.5903	0.1246	ns	>0.9999	0.9675	0.117	ns	>0.9999
**Nd**	0.5295	0.1398	ns	>0.9999	1.018	0.03666	ns	>0.9999
**Sm**	0.5017	0.1383	ns	>0.9999	1.017	0.04104	ns	>0.9999
**Eu**	0.4269	0.1556	ns	>0.9999	0.991	0.06139	ns	>0.9999
**Gd**	0.4139	0.2099	ns	>0.9999	0.9746	0.1213	ns	>0.9999
**Tb**	0.6033	0.1956	ns	>0.9999	0.9793	0.06153	ns	>0.9999
**Dy**	0.4647	0.2074	ns	>0.9999	0.9859	0.09458	ns	>0.9999
**Ho**	0.4312	0.1786	ns	>0.9999	0.9479	0.0656	ns	0.1063
**Er**	0.4503	0.2155	ns	>0.9999	0.9874	0.09051	ns	>0.9999
**Tm**	0.3586	0.1195	ns	0.1585	0.9484	0.08062	ns	0.3778
**Yb**	0.4585	0.2307	ns	>0.9999	0.9397	0.1231	ns	0.3909
**Lu**	0.4063	0.1724	ns	>0.9999	0.9815	0.07113	ns	>0.9999
**Y**	0.4436	0.1329	ns	>0.9999	0.9871	0.06922	ns	>0.9999
**Sc**	0.5491	0.3049	ns	>0.9999	1.01	0.05224	ns	>0.9999

Table S17. Ranking index of zebrafish larvae based on heatmap numeric differences.

Compounds	Behavioral Numeric Differences																			Total Numeric Differences
**Ce**	1	0.6	0.31	1.12	1.13	1.07	0.68	0.02	0.79	1.22	2.54	0.5	1.57	1.63	2.56	1	2.14	3.02	2.14	25.04
**Dy**	0.17	0.3	0.48	0.5	0.04	0.46	0.15	0.28	0.28	1.85	1.14	1.88	1.99	0.32	1.38	2.9	3.86	3.22	3.58	24.78
**Zr**	2.47	2.24	1.95	1.16	0.95	0.21	1.67	1.46	2.35	1.31	0.78	1.14	0.28	0.47	1.5	0.98	0.5	1.16	1.67	24.25
**Sr**	1.36	1.65	1.67	2.13	0.74	1.38	0.82	1.18	1.47	0.93	0.82	0.73	0.13	0.81	0.56	0.69	1.05	1.19	0.81	20.12
**Bi**	1.48	0.29	1.12	0.79	0.97	0.7	1.35	0.98	1.14	2.11	2.27	3.42	0.69	0.2	0.42	0.28	0.9	0	0.83	19.94
**Ni**	1.09	0.49	1.02	2.06	2.09	2.45	0.45	0.45	0.72	0.56	0.09	0.72	0.79	0.99	1.52	0.45	1.12	0.8	0.57	18.43
**Co**	0.16	0.28	0.36	0.54	0.28	0.91	0.18	0.57	0.1	2.33	0.93	2.45	1.35	0.72	1.2	1.56	0.8	1.45	1.89	18.06
**Zn**	1.34	0.63	0.05	1.48	0.83	1.4	0.66	0.02	0.95	1.48	1.25	0.84	1.19	1.54	0.61	0.22	1.15	1.25	0.67	17.56
**Pt**	1.84	1.51	1.43	0.57	1.56	0.98	1.57	1.23	1.62	1.06	0.35	0.43	0.9	0.09	0.13	0.03	0.9	0.37	0.28	16.85
**Sn**	1.15	1.55	1.59	1.48	1.95	1.23	1.18	1.13	1.43	1.02	0.3	0.08	0.44	0.36	0.21	0.52	0.2	0.37	0.62	16.81
**Gd**	0.81	1.44	1.37	1.29	0.12	0.09	1.72	1.44	1.31	0.71	0.79	0.48	0.35	1.32	1.47	0.49	0.37	0.29	0.41	16.27
**Ho**	1.01	0.7	0.96	0.26	1.56	0.63	1.72	1.56	0.32	1.27	0.65	0.65	1.39	0.17	0.93	0.74	0.17	0.2	0.05	14.94
**Cr**	0.72	0.6	0.18	1.07	0.47	2.05	1.31	0.16	0.76	0.84	1.35	0.16	2.53	0.53	0.26	0.14	0.65	0.67	0.42	14.87
**La**	0.34	0.67	0.91	0.9	1.43	1.55	0.49	1.27	0.53	0.16	0.28	0.92	0.13	1.38	0.13	1.62	0.94	0.34	0.8	14.79
**Pr**	0.82	1.07	1.13	0.88	1.62	0.39	0.23	1.01	1.01	0.41	1.2	1.04	0.83	0.91	0.48	1.3	0.24	0.03	0.14	14.74
**Mo**	0.69	0.97	0.98	1.2	0.91	1.38	1.01	0.98	0.83	0.95	0.36	0.11	0.49	0.53	0.39	0.72	0.81	0.56	0.15	14.02

**Tm**	1.04	1.12	0.93	0.69	0.25	0.33	1.26	1.81	1.15	0.09	0.86	0.26	0.52	0.34	0.62	0.63	0.45	1.19	0.31	13.85
**Sm**	0.82	0.95	1.09	1.23	1.29	0.23	0.73	1.35	0.95	0.11	1.33	0.87	0.69	0.51	0.21	0.92	0.05	0.03	0.19	13.55
**Mn**	0.89	0.41	0.24	1.29	0.01	0.15	0.61	0.24	0.66	1.23	0.93	0.96	0.42	1.13	1.21	0.95	0.98	0.62	0.54	13.47
**Yb**	1.39	0.96	1.04	0.1	1.28	0.13	1.55	1.3	1.1	0.64	0.14	0.06	1.33	0.43	0.65	0.31	0.22	0.42	0.24	13.29
**V**	0.96	1.12	1.25	0.21	1.08	0.25	1.25	1.09	1.07	0.03	0.52	0.37	0.85	0.05	1.94	0.32	0.37	0.2	0.17	13.1
**Tb**	0.47	0.52	0.81	0.38	0.48	0.98	0.38	0.52	0.3	0.38	0.48	0.55	0.25	1.91	0.32	0.99	1.39	0.6	1.22	12.93
**Nd**	0.2	0.77	1.03	0.5	0.43	0.26	0.11	0.49	0.64	0.6	1.3	0.83	1.14	1.36	0.91	0.98	0.38	0.31	0.52	12.76
**Li**	0.16	0.65	1.44	0.95	0.07	1.12	0.09	0.92	0.34	1.3	0.69	0.59	0.96	1.67	0.74	0.05	0.49	0.42	0.06	12.71
**Eu**	0.47	1	0.71	0.57	0.78	1.12	0.28	0.61	0.85	0.61	0.23	0.41	0.35	1.38	0.83	0.85	0.4	0.35	0.51	12.31
**Lu**	0.76	0.61	0.89	1.33	0.75	0.97	1.82	0.71	0.67	0.2	1.34	0.02	0.18	0.02	0.5	0.25	0.15	0.56	0.08	11.81
**Sc**	0.38	0.89	0.78	0.37	0.27	0.55	0.63	1.48	0.84	0.09	1.08	1	0.32	1.2	0.2	1.17	0.29	0.06	0.06	11.66
**Y**	0.52	0.57	0.44	0.7	1.39	1.64	0.28	0.9	0.55	0.44	0.16	0.45	0.92	0.56	0.94	0.32	0.2	0.3	0.03	11.31
**Al**	0.15	0.69	0.49	0.66	0.54	0.12	0.46	0.93	0.49	0.66	0.76	0.43	0.01	0.85	0.31	1.38	0.59	0.65	0.75	10.92
**Sb**	0.27	0.18	0.51	0.29	0.46	0.01	0.49	0.63	0.01	0.22	0.97	0.43	1.96	0.81	0.39	0.81	0.88	0.77	0.49	10.58
**Ga**	0.35	0.54	0.63	0.86	0.15	0.22	0.3	0.89	0.34	0.19	0.51	0.69	0.5	0.93	1.43	1.41	0.11	0.2	0.24	10.49
**Fe**	0.58	0.05	0.79	0.79	0.07	0.93	0.41	0.44	0.3	0.28	0.26	0.05	0.7	1.75	1.55	0.01	0.11	0.4	0	9.47
**Er**	0.62	1.09	0.85	0.25	0.04	0.16	0.8	0.32	1.11	0.16	0.04	0.39	0.06	0.94	0.11	0.33	0.19	0.21	0.03	7.7

Table S18. Ranking index of *Daphnia magna* based on heatmap numeric differences.

Compounds	Behavioral Numeric Differences																			Total Numeric Differences
**Mo**	0.22	0.62	1.88	2.61	2.83	0.53	3.18	0.6	0.09	0.73	3.89	1.41	0.75	1.23	0.19	0.13	0.68	1.07	1.11	23.75
**Mn**	1.04	0.34	1.31	1.46	0.68	1.41	1.01	1.81	2.93	0.79	3.04	1.74	1.13	1.3	0.11	0.07	0.9	0.67	0.64	22.38
**Sm**	0.36	1.18	0.93	1.57	1.4	1.64	1.56	1.03	1.26	0.88	0.68	0.94	1.08	0.93	1.17	1.13	1.54	1.5	1.48	22.26
**Tm**	1.91	1.68	0.95	0.85	1.64	0.11	1.43	0.72	1.36	1.07	0.66	0.9	1.06	0.89	1.11	1.08	0.84	0.74	0.69	19.69
**Nd**	0.81	1.22	0.64	0.97	1.06	0.74	0.86	1.45	0.8	0.55	0.67	0.94	1.09	0.93	1.13	1.1	1.42	1.41	1.4	19.19
**Yb**	0.32	2.04	0.43	0.11	1.21	2.26	1.4	0.46	0.01	1.76	0.66	0.6	0.79	0.61	1.03	1.04	1.43	1.39	1.4	18.95
**V**	0.96	0.65	2.74	2.07	0.12	1.22	0.48	0.88	0.97	0.06	0.19	0.06	1.84	2.02	1.86	1.83	0.31	0.26	0.28	18.8
**Sc**	1.12	0.89	0.04	0.66	0.36	1.29	0.38	0.99	1.21	2.25	0.67	0.9	1.08	0.92	1.15	1.11	1.32	1.17	1.15	18.66
**Gd**	1.03	0.59	0.36	0.36	0.65	0.9	0.58	1.47	1.01	2.66	0.6	0.85	1.02	0.88	0.89	0.91	1.14	1.24	1.25	18.39
**Sr**	1.13	0.44	1.09	2.08	1.67	0.51	1.94	1.76	1.69	0.52	0.26	1.33	0.12	0.27	0.79	0.66	0.94	0.49	0.46	18.15
**Pr**	1.77	0.88	1.01	0.16	0.41	1.28	0.13	1.19	0.32	1.88	0.65	0.93	1.1	0.94	1.11	1.08	1.11	0.96	0.93	17.84
**Ce**	0.88	1.84	1.92	1.73	0.54	1.73	0.95	0.6	0.97	0.9	0.54	0.81	0.92	0.78	0.72	0.72	0.69	0.24	0.18	17.66
**Ga**	0.19	1.01	0.08	0.92	1.16	0.53	1.11	0.96	0.64	0.84	0.03	1.09	0.64	0.16	0.7	0.6	2.02	2.09	2.04	16.81
**Zn**	0.53	0.23	0.94	0.52	0.65	0.41	0.88	1.2	0.75	0.15	1.08	0.16	1.81	1.81	1.42	1.54	0.79	0.66	0.68	16.21
**Pt**	0.16	0.47	0.32	0.9	1.05	1.94	0.93	1.45	1.05	0.79	0.18	0.99	0.65	0.94	1.35	1.26	0.22	0.34	0.28	15.27
**Tb**	1.98	0.39	0.16	0.63	0.85	0.22	0.77	1.06	0.36	0.43	0.66	0.88	1.06	0.9	1.13	1.09	0	1.22	1.15	14.94

**Sb**	1.35	0.02	0.39	0.98	1.07	0.37	0.99	0.44	0.46	0.02	1	0.07	0.06	0.58	1.77	1.77	1.4	1.11	1.08	14.93
**Li**	1.38	0.76	1.4	0.4	0.42	0.47	0.05	0.49	0.33	1	0.06	1.68	0.83	1.39	0.9	0.78	0.43	1.02	1.09	14.88
**Ni**	0.04	1.35	0.36	0.46	0.77	0.24	0.81	0.88	2.31	0.21	0.68	1.59	0.95	1.36	1.09	0.99	0.21	0.29	0.29	14.88
**Lu**	1.15	0.3	0.93	0.14	0.92	0.27	0.72	1.32	0.9	0.09	0.61	0.83	0.99	0.83	0.85	0.88	0.79	1.07	1.05	14.64
**Co**	1.45	0.72	0.74	0.03	0.37	0.85	0.16	1.31	0.37	0.3	0.13	0.23	1.28	0.68	0.66	0.68	1.3	1.37	1.4	14.03
**Y**	0.56	0.07	1.11	0.38	1.33	0.02	1.09	0.42	0.33	0.22	0.66	0.59	0.81	0.64	1.09	1.06	1.32	1.14	1.09	13.93
**Fe**	1.23	0.18	0.06	0.19	0.51	0.04	0.46	0.28	0.19	1.41	0.2	1.43	1.35	1.86	0.55	0.64	1.14	0.68	0.69	13.09
**Ho**	0.76	1.7	0.25	0.33	0.87	0.04	0.89	0.46	0.61	0.13	0.56	0.89	1.05	0.9	0.89	0.93	0.49	0.57	0.51	12.83
**La**	0.55	0.17	0.38	0.15	0.15	0.92	0.02	0.02	0.03	1.02	0.53	0.79	0.98	0.81	0.91	0.86	1.35	1.18	1.47	12.29
**Dy**	0.23	0.12	0.98	0.35	0.26	0.83	0.51	1.15	0.8	0.24	0.46	0.68	0.92	0.77	0.68	0.71	0.71	0.82	0.84	12.06
**Bi**	0.41	2.16	1.1	0.01	0.53	1.17	0.3	1.11	0.3	0.31	0.18	0.75	0.52	0.6	0.12	0.14	0.57	0.59	0.63	11.5
**Al**	1.18	0.6	0.09	0.15	0.3	0.45	0.31	1.11	0.28	0.05	0.16	0.53	0.02	0.6	1.42	1.93	0.48	0.87	0.91	11.44
**Eu**	0.83	0.1	0.4	1.3	1.62	0.5	0.31	0.94	0.87	1.09	0.48	0.43	0.03	0.02	0.46	0.47	0.15	0.42	0.4	10.82
**Sn**	0.19	1.18	0.47	0.56	0.42	0.06	0.56	0.61	0.81	0.31	0.01	0.38	0.15	0.27	1.05	0.92	0.59	1.1	1.12	10.76
**Zr**	1.33	0.85	1.62	1.02	0.02	0.54	0.43	0.23	1.11	1.45	0.15	0.18	0.23	0.48	0.23	0.22	0.05	0.27	0.18	10.59
**Cr**	0.54	1.18	0.73	0.59	0.01	0.58	0.18	0.43	0.17	0.21	0.02	0.4	0.17	0.68	0.21	0.17	1.17	1.23	1.11	9.78
**Er**	0.45	0.05	0.12	0.38	0.12	2.01	0.06	0.53	0.35	0.61	0.33	0.74	0.08	0.04	0.5	0.46	0.34	0.52	0.59	8.28
